# Tailoring the Swelling‐Shrinkable Behavior of Hydrogels for Biomedical Applications

**DOI:** 10.1002/advs.202303326

**Published:** 2023-08-06

**Authors:** Wenjun Feng, Zhengke Wang

**Affiliations:** ^1^ MOE Key Laboratory of Macromolecular Synthesis and Functionalization Department of Polymer Science and Engineering Zhejiang University Hangzhou 310058 China

**Keywords:** biomedical applications, high‐swelling hydrogels, hydrogels, non‐swelling hydrogels, shrinkable hydrogels

## Abstract

Hydrogels with tailor‐made swelling‐shrinkable properties have aroused considerable interest in numerous biomedical domains. For example, as swelling is a key issue for blood and wound extrudates absorption, the transference of nutrients and metabolites, as well as drug diffusion and release, hydrogels with high swelling capacity have been widely applicated in full‐thickness skin wound healing and tissue regeneration, and drug delivery. Nevertheless, in the fields of tissue adhesives and internal soft‐tissue wound healing, and bioelectronics, non‐swelling hydrogels play very important functions owing to their stable macroscopic dimension and physical performance in physiological environment. Moreover, the negative swelling behavior (i.e., shrinkage) of hydrogels can be exploited to drive noninvasive wound closure, and achieve resolution enhancement of hydrogel scaffolds. In addition, it can help push out the entrapped drugs, thus promote drug release. However, there still has not been a general review of the constructions and biomedical applications of hydrogels from the viewpoint of swelling‐shrinkable properties. Therefore, this review summarizes the tactics employed so far in tailoring the swelling‐shrinkable properties of hydrogels and their biomedical applications. And a relatively comprehensive understanding of the current progress and future challenge of the hydrogels with different swelling‐shrinkable features is provided for potential clinical translations.

## Graphical abstract

The current progress of high‐swelling hydrogels, non‐swelling hydrogels, and shrinkable hydrogels are summarized. These hydrogels are systematically introduced on the basis of the tactics employed so far in tailoring the swelling behavior of hydrogels and their practicable biomedical areas. Future challenges for clinical uses of these hydrogels are discussed as well.

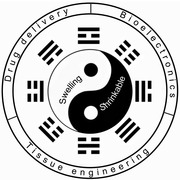

## 1 Introduction

Hydrogels have been the subject of extensive research in a multifaceted range of biomedical fields. They are 3D cross‐linked materials that are capable of retaining a significant fraction of water.^[^ ^1^ ^]^ The large water content and porous structure endow the hydrogels a degree of softness resemble natural living tissues,^[^ ^2^ ^]^ and permeability to various chemical and biological molecules.^[^ ^3^ ^]^ What's more, they are highly biocompatible,^[^ ^4^ ^]^ which will not impair the function of biomolecules and cells being encapsulated within or in direct contact with the hydrogel.^[^ ^5^ ^]^

But beyond that, an important characteristic of hydrogels, is their swelling properties in aqueous environments. Basically, the swelling behaviors of hydrogels are determined by the pore size on the hydrogel surface, the intermolecular spaces inside hydrogel network structures, and the hydrophilic and hydrophobic performance of the hydrogel.^[^ ^6^ ^]^ Under the comprehensive effect of these factors, hydrogels may exhibit different swelling behaviors in water. For example, hydrogels with large pores and a great deal of hydrophilic functional groups will swell to a great extent and imbibe water many times their own weights.^[^ ^7^ ^]^ In contrast, hydrogels with high crosslinking density and/or hydrophobicity will barely swell in liquid, that is, own non‐swelling nature.^[^ ^8^ ^]^ Besides, some stimuli‐responsive hydrogels can reversibly shrink with changes in external conditions, for example, temperature,^[^ ^9^ ^]^ and pH.^[^ ^10^ ^]^ The three types hydrogels can be respectively defined as high‐swelling hydrogels, non‐swelling hydrogels, and shrinkable hydrogels.

Of course, hydrogels with different swelling behaviors will have distinct physical properties, and therefore they are applicable to different biomedical scenarios. For example, high‐swelling hydrogels have mainly gained popularity in full‐thickness skin wound healing and tissue regeneration, and drug delivery over years due to that swelling ability is beneficial for absorbing blood and body fluid, transferring nutrients and metabolites, as well as diffusing and releasing drugs. Nevertheless, swelling in physiological conditions will inevitably cause obvious macroscopic deformation, and dramatically deteriorate the physical performance of the hydrogels, such as mechanical strength, tissue adhesion, and conductivity. Thus, non‐swelling hydrogels have served their functions extremely well in filling and repairing the vacant spaces of some internal vulnerable organs or tissues, use as bioadhesive sealants in surgical operations, bioelectronic devices, and so forth. Moreover, the volume shrinkage of hydrogels can actively drive noninvasive wound closure through exerting a centripetal force and enhance wound healing, and help achieve resolution enhancement of hydrogel‐based scaffolds for tissue regeneration. Besides, the shrinkage behavior can also be employed to accelerate drug release by squeezing out the entrapped biomolecules.

Nevertheless, to our knowledge, there still has been no literatures comprehensively summarizing the hydrogels from the perspective of swelling properties and their biomedical applications. Thus, it prompts us to give an overview on the hydrogels with different swelling features. The degree of swelling/deswelling of the as‐prepared hydrogels is often quantitatively indicated by swelling ratio (SR), which includes the weight swelling ratio and the volume swelling ratio.^[^ ^11^ ^]^ Due to that the SRs of high‐swelling, non‐swelling and shrinkable hydrogels are not well‐defined in previous literatures, this review provides practical guides for high‐swelling hydrogels with an equilibrium SR of more than 150%, non‐swelling hydrogels in the range of 0–150% and shrinkable hydrogels < 0% based on the relevant publications in the past five years (**Table** **1**). Current limitations and challenges of these hydrogels for clinical use are discussed as well. Therefore, we are fully confident that this review will gain readers greater insight into customizing the swelling properties of hydrogels for biomedical applications, and provide a clue to drive further development.

**Table 1:** Brief summary of hydrogels with various swelling features

Hydrogel types	Swelling ratios	Merits	Demerits	Fabrications	Biomedical applications	Refs.
High‐swelling hydrogels	>150%	High wound exudates absorption capacity, high cell recruitment and migration, high drug entrapment, diffusion and release	Macroscopic volume expansion, poor mechanical stability, weakened bioadhesion and conductivity, risk of tissue compression damage	Chemical modification, hydrophilic polymer self‐crosslinking, adding small molecule cross‐linkers	Tissue engineering, drug delivery	[12]
Non‐swelling hydrogels	0–150%	Excellent dimensional stability, long‐term wet‐adhesion performance, persistent mechanical strength and conductivity	Low drug loading and release efficiency, cannot exchange nutrients and bioactive molecules, low cell attachment, complex cross‐linked network design process	Functional polymer self‐crosslinking, synthesizing amphiphilic copolymer, polymer self‐assembling, adding small molecule cross‐linkers, solvent exchange, surface hydrophobic grafting	Tissue engineering, bioelectronics	[13]
Shrinkable hydrogels	<0%	Stimuli‐responsive, mechanically active, dynamic stiffening and contraction, promoted cell attachment and condensation, remotely controlled drug release	Risk of tissue tension damage, complicated molecular structure regulation process	Synthesizing stimuli‐responsive copolymer, introducing a second polymer, adding small molecule cross‐linkers	Tissue engineering, drug delivery	[14]

## 2 High‐Swelling Hydrogels

High‐swelling hydrogels (HSHs) will swell rapidly when in contact with aqueous solutions. It can absorb and hold massive aqueous fluids within their 3D porous structure without dissolution.^[^ ^15^ ^]^ This is primarily stemming from the low hydrogel network crosslink density and great quantities of hydrophilic groups (e.g., amino, hydroxyl, and carboxyl) on the macromolecular skeletons.^[^ ^1^, ^2^, ^16^ ^]^ The swelling capacity is a very significant property for advanced biomedical settings such as tissue engineering, drug delivery and controlled release. Judging from the scientific achievements so far, HSHs can be constructed based on natural polymers, synthetic polymers or mixtures of both. Their properties and biomedical applications will be introduced in this part (**Table** **2**).

**Table 2:** Brief summary of the constructions, properties, and biomedical applications of HSHs

Constructions	Hydrogel systems	Maximum SRs	Properties	Biomedical applications	Ref.
Based on natural polymers	CMCS, BTA, di‐amino Jeffamine	7560%	pH‐sensitive swelling, self‐healing, antibacterial	Not specific	[30]
	Gentamicin‐conjugated alginate	4962%	Adhesive, antibacterial	Not involved	[40]
	Oxidized HPC, CS	3570%	Self‐healing, injectable	Not specific	[12a]
	HA, chondroitin sulfate	1200%	Self‐healing, injectable, noncytotoxic	Bioprinting	[59]
	HA‐DA, rGO@PDA	300%	Injectable, self‐healing, photothermal, adhesive, conductive, hemostatic, antioxidant, antibacterial, sustained drug release	Wound healing	[12d]
	Gelatin, HP‐β‐CD, BMSC	198%	Injectable, biocompatible, biodegradable, osteogenic, angiogenic	Bone regeneration	[12e]
	l‐glutamic acid‐*g*‐CS, DOX	426%	pH‐responsive swelling, biocompatible, biodegradable, sustained drug release	Anti‐cancer drug delivery	[125]
	SA, gum tragacanth, phenolic compounds	300%	pH‐responsive swelling, biodegradable, sustained release	Intestine‐targeted drug delivery	[131]
Based on synthetic polymers	PAM, MPTAC, MBA	25 000%	Transparent, robust, elastic	Not involved	[12b]
	Carbopol934‐*g*‐PAA, EGDMA, diclofenac sodium	800%	pH‐responsive swelling, pH‐dependent drug release	pH‐dependent drug delivery	[133]
Blending natural and synthetic polymers	PAM, CS, SA	1258%	Long‐term mechanical stability and gastric retention, biocompatibility, biodegradable	Long‐term drug delivery	[12c]
	QCS, Matrigel, PAM	1200%	Stretchable, adhesive, biocompatible, antibacterial, hemostatic	Wound healing	[86]
	Alginate‐Nb, PEG‐Tz, DOX	2600%	Injectability, reduction‐responsive, biocompatible, remarkable drug loading efficiency, antitumor	Reduction‐responsive drug release	[138]

### 2.1 Based on Natural Polymers

HSHs made from hydrophilic natural biopolymers (e.g., polysaccharides and proteins) have many advantages, such as high water absorption capacity, biocompatibility, biodegradability, biological activity, and low cost.^[^ ^17^ ^]^ Besides, the presence of abundant reactive functional groups on the backbones of biopolymers can provide multifarious highly selective coupling points,^[^ ^18^ ^]^ facilitating the formation of HSHs. These fantastic features endow them immense superiority in biological areas. Amid a magnitude of biopolymers, chitosan, alginate, cellulose, starch, hyaluronic acid, etc., have been frequently utilized.

#### 2.1.1 Chitosan

Chitosan (CS), isolated from shells of marine crustaceans, etc.,^[^ ^19^ ^]^ is considered as one of the most important and abundant natural polymers on Earth. It is also the only linear alkalescent polysaccharide, which is constituted by irregularly arranged β‐(1,4)‐linked 2‐amino‐2‐deoxy‐d‐glucopyranose and β‐(1,4)‐linked 2‐acetamido‐2‐deoxy‐d‐glucopyranose units.^[^ ^20^ ^]^ The protonation of lateral amino groups under acid environment empowers CS to adhere to the negatively charged cytoderm of bacteria, causing membrane permeabilization and intracellular fluids release.^[^ ^21^ ^]^ Hence, CS have good antimicrobial activities against a board spectrum of microorganisms such as bacteria, fungi, and even some viruses.^[^ ^22^ ^]^ Beyond that, CS also exhibits good anti‐inflammatory,^[^ ^23^ ^]^ and hemostatic properties by promoting erythrocyte aggregation and platelet adhesion for blood clots formation.^[^ ^24^ ^]^ Nonetheless, the poor solubility and mechanical property of CS bring inconvenience to its practical applications.^[^ ^25^ ^]^ Many scientists have overcome the defects through chemical modification, or introducing nanoparticles.^[^ ^26^ ^]^ After amendment, the CS can be crosslinked via covalent bonds, supramolecular interactions, or a combination of both to build a hydrogel network structure with more hydrophilicity, higher swelling capacity, and mechanical stability.^[^ ^27^ ^]^ Therefore, CS has attracted much attention in developing HSHs with multiple biological properties.

In recent years, an indispensable strategy for preparing CS‐based HSHs is covalent crosslinking of CS or its derivatives with other biopolymers by virtue of Schiff base reaction. Schiff base is a dynamic imine linkage formed between amine and carbonyl groups, which can reversibly break and recombine.^[^ ^28^ ^]^ Hence, Schiff base based‐hydrogels own excellent viscoelasticity,^[^ ^29^ ^]^ and self‐healing ability.^[^ ^30^ ^]^ On these grounds, Maroufi et al. developed antibacterial CS‐based HSHs via the formation of Schiff base between modified dialdehyde guar gum and CS.^[^ ^31^ ^]^ Yu et al. reported a green strategy exploiting imine linkages for developing dynamic HSHs likewise. Differently, a water‐soluble CS derivative, carboxymethyl CS (CMCS), was crosslinked with benzene‐1, 3, 5‐tricarbaldehyde, whose hydrophilicity was improved by reacting with di‐amino Jeffamine. The amino and carboxyl groups along CMCS chains endowed the hydrogels strongly pH‐sensitive swelling behaviors. As a result, maximal SR was observed at pH 8, reaching 7560%. And the combination of positive‐charged amino groups and active Schiff bases containing aromatic ring was proved to impart the hydrogels brilliant antibacterial activity against *E. coli*.^[^ ^30^ ^]^ At an advantage, these preparation methods avoided potentially toxic small‐molecule crosslinkers.

**Figure 1:**
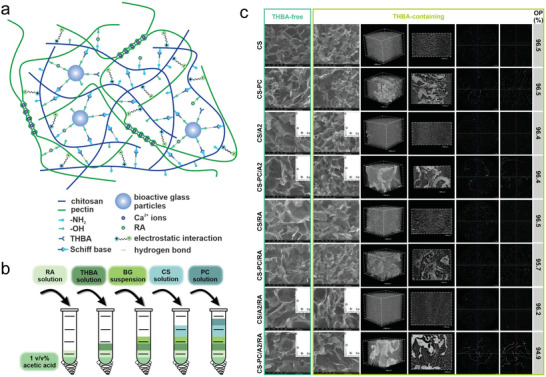
Synthesis and characterization of HSHs based on natural polymer. a) Illustration of the CS‐PC/BG/RA hydrogel network. b) Scheme of the adding order of the ingredients. c) SEM images and EDX spectra of the THBA‐free and THBA‐containing hydrogels. Representative µCT analyses of 3D reconstructions, cross sections, and open porosity (OP) of the THBA‐containing hydrogels. Adapted with permission.^[^ ^33^ ^]^ Copyright 2022, The Authors. Published by Elsevier.

In addition, the electrostatic interaction between the positively charged CS and anionic compound, has been used to construct CS‐based HSHs as well. For instance, the electrostatic contacts between CS and poly(γ‐glutamic acid)(γ‐PGA) yielded a porous hydrogel with a high swelling capacity of 1398%.^[^ ^6b^ ^]^ Situ's group developed different ionically crosslinked CS hydrogels by using different anionic crosslinkers, that is, sodium hexametaphosphate and sodium tripolyphosphate. In comparison, hydrogels crosslinked by sodium tripolyphosphate had better swellability and biocompatibility.^[^ ^32^ ^]^ Particularly interesting is that CS‐based hydrogels were first crosslinked with 2, 3, 4‐trihydroxybenzaldehyde (THBA), and modified with pectin (PC), bioactive glass (BG), and rosmarinic acid (RA) (**Figure** **1**a), leading to enhanced mechanical strength, high swelling ability and delayed degradation. Benefiting from the adding order (Figure 1b), the CS‐PC/BG/RA hydrogels were relieved of BG agglomeration observed in PC/BG hydrogels (Figure 1c). And the synergetic advantages of the incorporated bioactive substances imparted the prepared hydrogels high antioxidant activity, anticancer activities, and ability to mineralize in simulated body fluid.^[^ ^33^ ^]^

#### 2.1.2 Alginate

Alginate is a linear anionic polysaccharide primarily extracted from brown algae.^[^ ^34^ ^]^ It is a copolymer of α−1,4‐l‐guluronic acid (G) and β−1,4‐d‐mannuronic acid (M) units in either repeating or alternating sequence, linked by β−1,4‐glycosidic bonds.^[^ ^35^ ^]^ In various studies, alginates have been demonstrated to lower proinflammatory cytokines concentration and inhibit free radical formation,^[^ ^36^ ^]^ activate macrophages,^[^ ^37^ ^]^ increase collagen I deposition, and promote re‐epithelization.^[^ ^38^ ^]^ Therefore, this biocompatible biopolymer is highly attractive for clinical applications. And alginate hydrogel formation usually occurs through the ionic association between G units and divalent cations, such as Ca^2+^.^[^ ^39^ ^]^

Alginate hydrogels have long driven the research and development of HSHs based on natural polymers. For example, microbicidal alginate hydrogels achieved through reacting with antibiotic gentamicin sulfate mediated by carbodiimide chemistry, disclosed excellent SR (4962%) and antibacterial effect against *S. aureus*. and *E. coli*.^[^ ^40^ ^]^ In another study, sodium alginate (SA) hydrogel matrix was imparted with anticancer and tissue regeneration activities through functionalized with graphene oxide (GO). The resulted multifunctional hydrogels could swell and release silver sulfadiazine under different pH media.^[^ ^41^ ^]^ Anitua et al. reported three kinds of hydrogel bioinks with high swellability through combining alginate and gelatin (GA), enriching either hydroxyapatite (GAHA) or hydroxyapatite and Plasma Rich in Growth Factor (GAHAP). The hydroxyapatite‐contained GAHA and GAHAP hydrogels showed higher mechanical strength, proliferative rate, and osteogenic differentiation, while Plasma Rich in Growth Factor significantly enhanced cell adhesion and chemotaxis.^[^ ^42^ ^]^

#### 2.1.3 Cellulose

Another natural polymer extensively explored for developing HSHs is cellulose. Predominantly constituted by β‐d‐glucose units linked through 1,4‐β‐glycosidic bonds, cellulose is ranked as the most profuse and nearly inexhaustible polysaccharide in natural sources.^[^ ^43^ ^]^ Thus, it has many merits including but not limited to low price, biocompatibility and biodegradability.^[^ ^44^ ^]^ The abundant hydrophilic hydroxyl groups on cellulose chains create many opportunities for designing cellulose‐based hydrogels by chemical bonds and electrostatic adsorption.^[^ ^45^ ^]^ Nevertheless, in virtue of the strong intra‐ and inter‐molecular hydrogen bonds deriving from the absence of side chains, cellulose chains feature many ordered structures (i.e., crystalline regions).^[^ ^46^ ^]^ This characteristic make cellulose hydrogels can hardly swell in water.^[^ ^47^ ^]^ Chemical treatment by grafting or functionalization of the cellulose surface is an attractive measure to damage the hierarchical structure,^[^ ^48^ ^]^ and increase the number of hydrophilic groups exposed to water molecules,^[^ ^49^ ^]^ thereby overcome the limitation and develop new desired characteristics. As a result, cellulose derivatives, mainly including carboxymethyl cellulose (CMC) and hydroxypropyl cellulose (HPC), have more attractive in HSHs scenes compared to cellulose.

To this end, Edgar's group designed and prepared a family of oxidized HPC/CS hydrogels,^[^ ^12a^ ^]^ oxidized HPC/Jeffamine hydrogels,^[^ ^50^ ^]^ which were crosslinked by Schiff base. Both type hydrogels exhibited high SRs, tunable mechanical modulus, and rapid self‐healing ability. Thanks to the Jeffamine crosslinker, the oxidized HPC/Jeffamine hydrogels further displayed thermal responsivity. Peighambardoust's team and Huang's team grafted hydrophilic copolymers onto CMC and acquired hydrogels with enhanced equilibrium swelling. Concretely, the former type CMC hydrogels were synthesized by grafting copolymerization of acrylic acid (AA) and itaconic acid along the CMC backbone,^[^ ^51^ ^]^ while the latter were grafted with copolymers of AA and acrylamide (AM).^[^ ^52^ ^]^ Interestingly, the presence of carbon black nanoparticles could further enhance the swelling performance of the hydrogels, while GO could enhance the thermal stability.

#### 2.1.4 Starch

Amongst various types of biopolymers, starch is the main carbohydrate in maintaining human nutrition and health. It has the advantages of wide source, renewability, good biosecurity, and biodegradability.^[^ ^53^ ^]^ Starch is comprised of two glucose polymers: lightly branched amylose and highly branched amylopectin, which are linked by α−1, 4 and α−1, 6‐glycoside bonds.^[^ ^54^ ^]^ Similar to cellulose, the strong hydrogen bonding between the thousands of hydroxyl groups on starch chains leads to a rigid semi‐crystalline network, causing poor solubility and mechanical property.^[^ ^55^ ^]^ By introducing new groups on its chains, starch can be a very prospective biopolymer used in developing HSHs.

By means of acid‐base synergistic pretreatments, Chen and coworkers synthesized hydroxybutyl starch with the highest degree of substitution (DS). After copolymerization with *N*‐isopropylacrylamide (NIPAm), the hydroxybutyl starch yielded a series of temperature‐sensitive hydrogels with increased water absorption rate and equilibrium SR.^[^ ^56^ ^]^ Based on oxidized starch, Namazi's team prepared smart hydrogels with pH‐responsive swelling behaviors. The highest swelling was observed at pH 7 owing to the electrostatic repulsive forces between the carboxylate anions of oxidized starch. And the SR would decrease with the increase of salt concentration and valence of the cations. Furthermore, through introducing Zinc oxide nanoparticles (ZnO NPs), the hydrogels showed excellent antibacterial activities against *S. aureus* and *E. coli*.^[^ ^57^ ^]^ Another study developed a superabsorbent starch‐graft‐polyacrylic acid (PAA) hydrogel using cellulose nanofibers (CNF) as reinforcing material. After adding 5 wt% CNF, the SRs in NaCl, CaCl_2_, and AlCl_3_ solutions respectively showed an increment from 109 to 193, 62 to 110, and 56 to 99 (*g*_water_/*g*_hydrogel_), which was due to the reduced entanglement of polymer chains and interactions between the hydrophilic groups. Compressive strength and Young's modulus achieved 63.3 and 31.6 kPa, respectively corresponded to 1.69 and 2.40 times of unreinforced samples. Also, the surface of CNF reinforced hydrogel could better facilitate the proliferation of live cells.^[^ ^58^ ^]^

#### 2.1.5 Other Biopolymers

Also, there are some studies of HSHs based on other natural polymer materials, such as hyaluronic acid (HA) and gelatin. In the work of Mihajlovic et al., the combination of highly charged and hydrophilic chondroitin sulfate and HA yielded a viscoelastic double network (DN) hydrogel with significant swelling capacity (maximum SR ranging from 6 to 12). Integrating the short lifetime of dynamic hydrazone cross‐links and long‐lasting Diels‐Alder cross‐links, the formulated DN hydrogels simultaneously possessed easy processability and self‐healing features, and long‐term structural integrity. Moreover, they were noncytotoxic to mesenchymal stromal cells, and its degradation products would not affect the macrophages.^[^ ^59^ ^]^ Another previous research has synthesized gelatin‐based hydrogels which exhibited mechanical properties similar to soft human tissue (*G*’ = 1–100 kPa) and high swelling capabilities (1000–3000 vol%), via thiol‐ene Michael‐addition between methacrylated gelatin and oligo(ethylene glycol) dithiols.^[^ ^60^ ^]^

### 2.2 Based on Synthetic Polymers

Synthetic polymer hydrogels outperform natural polymer hydrogels in many aspects, especially for their better mechanical properties because most of them were crosslinked by strong covalent bonds.^[^ ^61^ ^]^ Their physicochemical properties can be much easier customized by adjusting chemical compositions, molecular weights, and block structures, in order to meet numerous specific requirements.^[^ ^35^, ^62^ ^]^ Typically, synthetic HSHs are polyelectrolyte hydrogels which have high osmotic pressure originated from plentiful dissociated counterions, and strong electrostatic repulsion between the ionized groups of polymer chains. And they are synthesized via free radical polymerization approach under an inert atmosphere.

**Figure 2:**
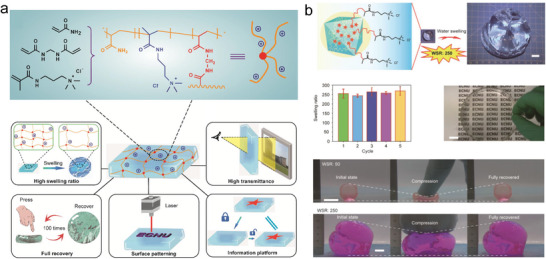
Synthesis and characterization of HSHs based on synthetic polymer. a,b) Representation of the synthetic polymer‐based cationic hydrogel with high water swelling ratio (WSR) and light transmittance after swollen, excellent mechanical property, surface patterning, and information camouflage and decryption. Scale bar: 1 cm. Adapted with permission.^[^ ^12b^ ^]^ Copyright 2022, American Chemical Society.

On this basis, Sorkhabi et al. constructed macroporous SiO_2_/PAA hydrogel with high SR (ranging from 500 to 4000) by in situ solution polymerization.^[^ ^63^ ^]^ Thakur et al. synthesized a itaconic acid grafted poly(acrylic acid‐co‐aniline) hydrogel with a high swelling percentage of 1755.3%.^[^ ^64^ ^]^ Cėpla et al. developed tens of nanometer‐thick hydrogel coatings containing methyl methacrylic acid, 2‐hydroxyethyl methacrylate (HEMA), and polyethylene glycol methacrylate (PEG_10_MA) monomers. Together with the SRs up to 3.2, the good stability and inhibition to nonspecific protein binding imparted the hydrogel great potential in mimicking extracellular matrix (ECM).^[^ ^65^ ^]^ Ayres's group prepared polyurethane‐based hydrogels with high SRs (300–2500%) owing to the hydrophilicity of lactose units. And the swelling degree and mechanical properties of the resulting hydrogels could be controlled by the chain length of polyethylene glycol (PEG).^[^ ^66^ ^]^ In another research, decomposable superabsorbent hydrogels were synthesized by modifying poly(allylamine hydrochloride) with a recombinant protein owning a specific thrombin recognition site. Although the recombinant protein was not a superabsorbent material like poly(allylamine hydrochloride), the hydrogel with 15% protein still maintained a high SR to 900%. And the degradability of the polyelectrolyte hydrogel was realized by adding specific enzyme thrombin at indicated time point.^[^ ^67^ ^]^ Particularly interesting was the work of Xu et al., in which a cationic monomer was copolymerized into polyacrylamide (PAM) networks (**Figure** **2**a). Advantageously, the hydrogel exhibited high SR and transmittance, as well as robust and elastic mechanical property, which enabled the camouflage of complex information on hydrogel surface by swelling, and decryption under white light (Figure 2a,b).^[^ ^12b^ ^]^

Nevertheless, chemically cross‐linked synthetic polyelectrolyte hydrogels are often subjected to high brittleness,^[^ ^68^ ^]^ thus restricting their widespread applications. To acquire considerable toughness, designing physically cross‐linked synthetic polyelectrolyte hydrogels or introducing a second physical network have been identified as preferable choices. On this ground, a series of hydrophobic associated poly(acrylic acid‐acrylamide‐lauryl methacrylate) [poly(AA‐AM‐LMA)] hydrogels were developed. On one hand, the macroporous network brought the hydrogels high water absorption capacity, which could imbibe water up to 1000 g g^−1^ without dissolution. On the other hand, the multiple physical interactions inside the hydrogel endowed it high stretchability of 1500–3000%, tensile strength of 50–150 kPa, and toughness up to 2.4 MJ m^−3^.^[^ ^69^ ^]^ In contrast, Dixit and Bag introduced a reversibly crosslinked polyvinyl alcohol (PVA)‐Borax network into permanently crosslinked poly(NIPAm‐*co*‐AM) network with temperature‐sensitivity and high swellability. Finally, the DN hydrogels indicated excellent maximum compressive stress of 5.75 MPa and fracture energy of 36.72 MJ m^−3^, and high stretchability up to 944% and tensile strength up to 0.473 MPa. And they also displayed desirable resilience, which could maintain their original shapes after many mechanical cycles.^[^ ^70^ ^]^

### 2.3 Based on Natural and Synthetic Polymers

The blend of biopolymers and synthetic polymers can result in a hybrid network integrating the intrinsic features of both kind polymers. Thus, the hybrid hydrogels will overcome the drawbacks of the individual components (e.g., poor mechanical stability of biopolymers, low biodegradability, and lack of biological activities of synthetic polymers). The superb comprehensive properties are beneficial for applying the hydrogels in biomedical sciences. Meanwhile, the synergistic cumulative effects of hydrophilicity can ease the realization of high swelling capacity. Therefore, there is a trend of developing HSHs through blending natural and synthetic polymers.

**Figure 3:**
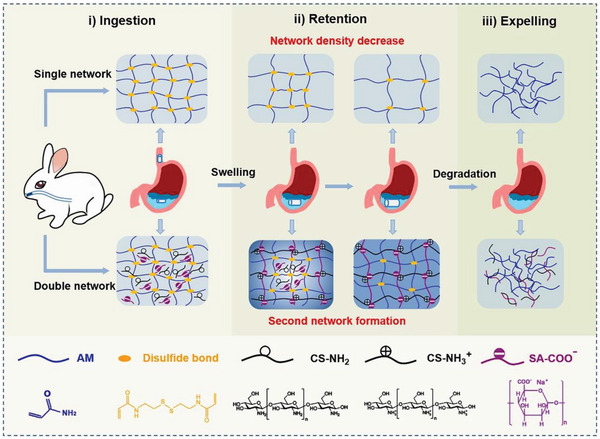
Synthesis of HSHs based on natural and synthetic polymer. The double network hybrid hydrogel can be ingested orally. Adapted with permission.^[^ ^12c^ ^]^ Copyright 2022, Elsevier Ltd.

The first related work comes from Popescu et al., which produced degradable alginate/poloxamer hydrogels via thiol‐acrylate photopolymerization between alginate equipped with thiol groups and acrylate‐modified poloxamer. The as‐prepared hydrogels had high swelling capacity (SR ranging between 6 and 13) in simulated physiological conditions. In vitro biological assays have confirmed that the hybrid hydrogels could facilitate human keratinocyte proliferation, and had an anti‐inflammatory effect on lipopolysaccharides‐activated keratinocytes.^[^ ^71^ ^]^ Particularly noteworthy is the approach for manufacturing dually cross‐linked (DC) and DN hybrid hydrogels integrating covalent and physical cross‐linking, which is superior in acquiring comprehensive mechanical properties.^[^ ^72^ ^]^ According to this, a series of chemically physically DC CS/PVA hydrogels were obtained. The hydrogels displayed high SRs in water (up to 92) and phosphate‐buffered saline (PBS) (up to 74). Although the elastic modulus was not particularly distinguished (3–30 kPa), the hydrogels were very flexible and elastic. Notably, in situ formation of silver nanoparticles (Ag NPs) enabled the CS/PVA hydrogels remarkably inhibit the growth of *S. aureus* and *K. pneumonia*. And the cell vitality tests proved the non‐cytotoxicity of these natural and synthetic polymer‐based hydrogel systems.^[^ ^73^ ^]^ Zhang's team prepared DN hybrid hydrogel with SR reaching 1258.1%. Interestingly, the formulated DN hydrogel could reside in stomach by the dramatic swelling of the first PAM network, and keep mechanical strength through gradually forming the second CS/SA network under the action of gastric fluid. After retention, the hydrogel can be biologically degraded and expelled from the body (**Figure** **3**).^[^ ^12c^ ^]^

### 2.4 Biomedical Applications

#### 2.4.1 Tissue Engineering and Regenerative Medicine

##### Wound Healing

Wound healing involves four integrated and overlapping phases: hemostasis, inflammation, proliferation, and remodeling/maturation.^[^ ^74^ ^]^ To deter impaired healing of the wound, these phases must progress in order with sufficient time.^[^ ^75^ ^]^ HSHs have many superiorities in speeding up the four phases. First, benefiting from their high swelling rate and water absorption ability, HSHs can quickly absorb the blood and extrudates of the wounds.^[^ ^76^ ^]^ Second, it can serve as physical barrier against external microorganisms, thereby reducing inflammation.^[^ ^77^ ^]^ Besides, the interconnected porous network of HSHs provides benefits in transmitting oxygen, exchanging nutrients, and bioactive molecules, and recruiting stem cells for fast wound repair and regeneration.^[^ ^78^ ^]^ And they can also maintain a comparable moist microenvironment on the wound bed,^[^ ^71^ ^]^ which promotes epidermal migration and tissue regeneration with less scarring.^[^ ^78^ ^]^ So, HSHs have a broad prospect in wound healing.

From the viewpoint of clinical practice, injectability is a crucial property as it facilitates administration with minimal invasion and adaptability to the complex contour of target sites.^[^ ^79^ ^]^ Hence, based on a dynamic sliding physical crosslinking mechanism, Zhu et al. prepared injectable self‐healing γ‐PGA‐Lysine hydrogels with a SR of 2750% in PBS and of nearly 10 000% in distilled water, which exhibited good capacity to accelerate wound closure and reconstruction. Through using 10% diethylene glycol as interstitial phases, the as‐prepared hydrogels demonstrated high stretchability with the maximum elongation of 2705%.^[^ ^80^ ^]^ In order to assist the healing of chronic wounds, Dong et al. prepared an injectable γ‐PGA hydrogel loaded with superoxide dismutase, a kind of antioxidant that can minimize reactive oxygen species production. The injectability of the hydrogel was attributed to the introduction of poly(*N*‐isopropylacrylamide) (PNIPAm). With the phase transformation temperature of 28.2 °C, the hydrogel could be injected in vivo and in situ form a rigid hydrogel. Together with high SR (980%) and good water retention property, the superoxide dismutase‐loaded hydrogel dressings exhibited best wound closure rate in diabetic rat skin defect model.^[^ ^81^ ^]^

Nowadays, bacterial infection containing multi‐resistant bacterial strains infection, is one of the most critical clinical concerns after tissue trauma.^[^ ^82^ ^]^ To earn a broad spectrum of strong antibacterial effects, scientists have explored many ingenious strategies, including chemically modifying the polymers, introducing metallic nanoparticles, carrying antibiotics, or in combination of them. Through taking full advantage of these methods, numerous outstanding antibacterial HSHs have been made for the recent years, which is highly desirable for producing a superior healing effect.

**Figure 4:**
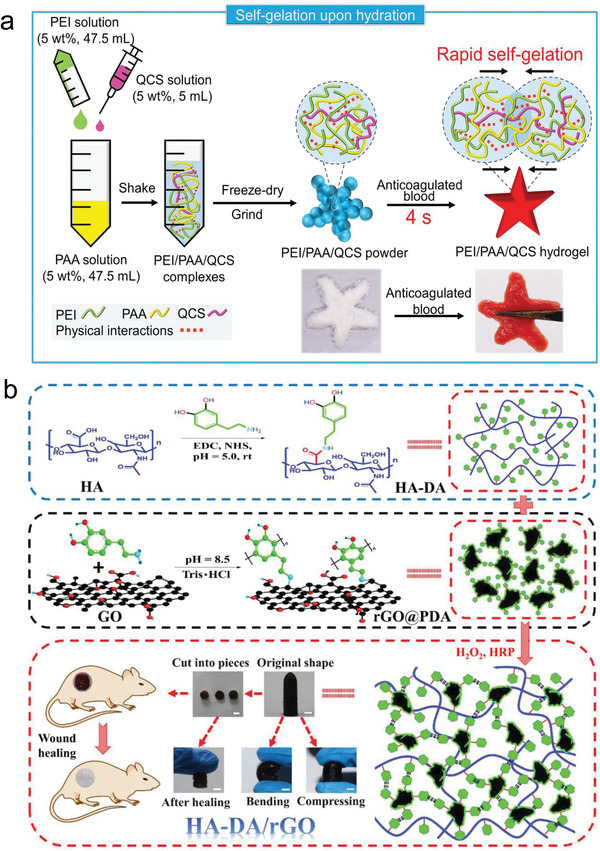
Exploiting the high‐swelling properties for hemostasis and full‐thickness skin wound healing. a) Fabrication diagram of PEI/PAA/QCS powder and the formation of PEI/PAA/QCS hydrogel after adding anticoagulated blood. Adapted with permission.^[^ ^88^ ^]^ Copyright 2021, Wiley‐VCH GmbH. b) Synthesis scheme and macroscopic performance of HA‐DA/rGO hydrogel applicated in wound healing. Scale bar: 5 mm. Adapted with permission.^[^ ^12d^ ^]^ Copyright 2019, Wiely‐VCH Verlag GmbH & Co. KGaA.

In the respects of chemical modification, introducing quaternary ammonium group into CS have been most extensively employed. It can not only enhance the inherent antimicrobial effects of CS by increasing the positive charges, but also greatly improve its water solubility.^[^ ^83^ ^]^ In addition, quaternized chitosan (QCS) can form multi‐interactions (electrostatic interactions, hydrogen bonding, etc.) with biological tissues, which guarantees certain adhesion behavior of hydrogel.^[^ ^84^ ^]^ The integration of antibacterial function and bioadhesiveness can ensure the injury site keep closed and combat bacterial infection.^[^ ^85^ ^]^ So several research groups have invented antibacterial HSHs based on QCS. Xue et al. designed a high‐swelling multi‐functional QCS‐Matrigel‐PAM hydrogel with a physical‐chemical DN structure. The equilibrium SR of the hydrogel reached about 1200%. In addition, the hydrogel showed antibacterial, hemostatic, excellent stretchable, suitably adhesive, and biocompatible properties. As a result, in a full‐thickness skin defect model, it significantly enhanced wound closure, collagen deposition, and promote regeneration of skin appendages.^[^ ^86^ ^]^ Being lyophilized or dried in advance, some hydrogel dressings with outstanding swelling ability still capable of in situ forming a hydrogel through absorbing the body fluid on the wound area. Therefore, these hydrogel dressings can be applicated in the shapes of a dry sponge, powder, and so on. The desiccation of these types wound dressings render it the capacity to swell more quickly and be preserved for a longer period of time. Specifically, in comparison with sponge, the powder can be more appliable to various wound surfaces.^[^ ^87^ ^]^ With this background, an antibacterial polyethyleneimine (PEI)/PAA/QCS powder, was designed and prepared by Bian and coworkers (**Figure** **4**a). After deposited on bleeding wounds, the powder could fastly transform into an adhesive hydrogel and aggregate blood cells and platelets. Thus, the PEI/PAA/QCS powder demonstrated rapid and effective hemostasis even against massive hemorrhage in non‐compressible porcine spleen and liver. Besides, it could expedite full‐thickness skin wound healing as well.^[^ ^88^ ^]^ Overall, both the above elucidated hydrogels with synergistic effect of antibacterial, hemostatic, and adhesive properties provide inspirations for clinical use as bioactive wound dressings. But for noncompressible visceral, and high‐pressure arterial bleeding wounds, there are still many challenges remain to be addressed for simultaneously achieving rapid and effective hemostasis and subsequent wound healing process. Especially in the peculiar pathophysiological abnormalities of diabetic wound environment, the wound treatments place higher demands on the antibacterial, antioxidant, angiogenesis, bioadhesiveness, swelling rate, as well as moisture retention property of the hydrogel dressings.^[^ ^89^ ^]^

Adding metallic nanoparticles, especially silver,^[^ ^90^ ^]^ or carrying antibiotics,^[^ ^91^ ^]^ is also a superb idea for eradicating local bacterial infection and keeping a sterile wound healing environment.^[^ ^26a^ ^]^ For example, through adding sodium borohydride to generate silver nanocomposite from silver nitrate and crosslink guar gum‐grafted‐polyacrylamidoglycolic acid polymer, a variety of injectable self‐healing HSHs were prepared and exhibited enhanced bacterial inactivation properties against wound pathogens.^[^ ^92^ ^]^ Notably, as famous antibacterial agents, the safety of Ag NPs and ZnO NPs are certificated by the Food and Drug Administration.^[^ ^14^, ^93^ ^]^ Furthermore, several research groups attempt to prepare antibacterial HSHs by delivering antibiotics. In one study, berberine was loaded into microbial transglutaminase and Ca^2+^ DC gelatin/SA hydrogels, which presented high SR over 9.99. The berberine‐carried hydrogels showed sustained drug release for 168 h and great antibacterial activity against *S. aureus*. Intriguingly, stemmed from the ethylene diamine tetraacetic acid‐induced detachment performances, the hydrogels could be painlessly detached from the wound.^[^ ^94^ ^]^ In another, a class of micelle‐crosslinked hydrogels with high swelling capacity (4688–10 753%) and good water vapor transmission rate, were synthesized for releasing ciprofloxacin and silver sulfadiazine. Significantly, the drug‐loaded hydrogels exhibited inhibitory effect against Gram‐negative *E. coli* and *P. aeruginosa* and Gram‐positive *S. aureus*.^[^ ^95^ ^]^ Notably, after encapsulating amoxicillin, a series of injectable hydrogel formed via Schiff base reaction between oxidized HA‐graft‐aniline tetramer (AT) and N‐carboxyethyl CS, revealed effective antibacterial property. The hydrogels themselves demonstrated high equilibrated SR from 23 to 36, as well as good in vitro biocompatibility and biodegradability. The existence of AT also imparted the amoxicillin‐loaded hydrogels conductivity and desirable antioxidant ability, which synergistically speeded up full‐thickness skin wound healing with thicker granulation tissue, higher collagen disposition, and more angiogenesis. The present approach provided a clue for designing promising electroactive injectable hydrogel wound dressings.^[^ ^96^ ^]^ But should not be ignored is that the antibiotic abuse may build up bacterial resistance.^[^ ^97^ ^]^

Over the years, photothermal therapy has attracted particular attention in fighting against common pathogens or antibiotic‐resistant ones.^[^ ^98^ ^]^ Therefore, it is meaningful for adding materials with photothermal activity into HSHs for wound healing. In one contribution, a suit of injectable photothermal antibacterial HSHs were developed based on hyaluronic acid‐dopamine (HA‐DA) and polydopamine‐coated‐reduced GO (rGO@PDA) (Figure 4b). The HA‐DA endowed the hydrogels with antioxidant, bioadhesive, and hemostatic capacities, while the incorporation of rGO@PDA promoted electrical conductivity and in vivo near‐infrared light (NIR) irradiation antibacterial behavior. Moreover, after loaded with doxycycline, the HA‐DA/rGO hydrogel also showed sustained drug release capacity more than 10 days, thus displayed best vascularization, granulation tissue thickness and collagen deposition in full‐thickness wound repair model.^[^ ^12d^ ^]^ The results above demonstrated that using a combined approach to promote antibacterial activity can contribute to a better therapeutic effect. But it is more suggested to utilize the methods which will not bring bacterial resistance.

All in all, the ongoing findings have clearly revealed lots of evidences on therapeutic efficacy of HSHs in speeding up full‐thickness skin wound healing. Nonetheless, the current investigations have not carried out comprehensive potential organ toxicity tests in mammals, and are limited to exploit single wound model.^[^ ^16^ ^]^ Moreover, the latent factors (e.g., stress around the wound edges) influencing wound healing are yet to be explored.

##### Other Tissue Regeneration

It is widely known that hydrogels possess a close similarity to natural ECM.^[^ ^99^ ^]^ And porous internal structure and high swelling behavior can powerfully support cell migration, the entrapment, diffusion, and release of nutrients and metabolites, thereby accelerating tissue regeneration.^[^ ^100^ ^]^ Therefore, HSHs have been diffusely applied as tissue engineering scaffolds.^[^ ^101^ ^]^

However, the mechanical stability of classic HSHs is poor, which seriously impeded their actual utilization.^[^ ^102^ ^]^ To address current bottlenecks, several groups have respectively prepared click, interpenetrating polymer network (IPN), and DN HSHs. The click hydrogel scaffold was obtained via the thiol‐acrylate photopolymerization of silk fibroin and thiol‐terminated PEG. This production approach acquired high DS of acrylate groups on HA backbone by employing dimethyl sulfoxide (DMSO) as solvent, and avoided oxygen inhibition effect. As a result, the hydrogel demonstrated improved stiff.^[^ ^103^ ^]^ Combing sonication and photocrosslinking approaches, IPN hydrogels with equilibrium SRs of above 6 were formed between gelatin methacrylate (GelMA) and silk fibroin (SF). Exhibiting excellent mechanical properties and resistance to enzymatic degradation, the SF‐GelMA IPN hydrogels are extremely favorable for load‐bearing tissue engineering. And it was affirmed that MC3T3‐E1 pre‐osteoblasts could growth on both surface and inside of the hydrogels.^[^ ^104^ ^]^ With maximum SR of 5312%, a DN hydrogel of gellan gum and poloxamer‐heparin demonstrated the potential as a cell vehicle for stem cell applied to tissue engineering by supporting the survival and retaining the morphology and phenotype of bone marrow‐derived stem cells (BMSCs) in vitro and in vivo.^[^ ^105^ ^]^

Equally significative is the publication dealing with the fabrication of injectable HSHs scaffolds. Utilizing hydroxypropyl‐β‐cyclodextrin (HP‐β‐CD) to crosslink gelatin via hydrogen bonds, Yuan et al. yielded hydrogels with good injectability, faster biodegradation and high swellability. After encapsulated into the hydrogels, BMSCs showed high cell viability (>90%) and more efficient osteogenic differentiation within 2 weeks than in the osteogenic medium. According to in vivo analysis, the injectable HP‐β‐CD‐gelatin/BMSC hydrogels effectively enhanced angiogenesis and osteogenesis of the necrosis site of the femoral head. In conclusion, the advanced injectable hydrogels offered a prospective way to improve femoral head necrosis bone repair.^[^ ^12e^ ^]^

Based on the facts that appropriate biological functionality is an important factor for promoting tissue regeneration, Malik et al. achieved CS/CMC/hydroxyapatite hydrogels with high SRs (2181.9–2856.6%) and enhanced angiogenesis through holding one of the vital hormones within human body, thyroxin.^[^ ^106^ ^]^ This further reflects another fantastic properties of HSHs, that is, top‐notch drug‐loading and releasing behavior, relying on which HSHs not only have superiorities in tissue engineering and regenerative medicine, but also drug delivery.

#### 2.4.2 Drug Delivery

HSHs with highly porous structures have the merits of efficiently encapsulating and releasing both hydrophilic and hydrophobic drugs, proteins, and peptides.^[^ ^107^ ^]^ This is due to the reason that the high swelling ability will strongly facilitate the solvent uptake and drug diffusion.^[^ ^108^ ^]^ In addition, they are highly biocompatible and biodegradable.^[^ ^76a^ ^]^ Therefore, HSHs are remarkable candidates for drug delivery and swelling‐controlled releasing. And the drug release profile is governed by the coupling of diffusion and macromolecular relaxation.^[^ ^109^ ^]^

In this context, Grimaudo et al. developed high swelling inserts of sodium hyaluronate and HP‐β‐CD using poly(ethylene glycol) diglycidyl ether (PEGDGE) as cross‐linker, to deliver cyclosporine to ocular surface.^[^ ^110^ ^]^ In order to mimic the composition of bone, Chung and co‐workers synthesized an oxidized HA/type I collagen hydrogel containing β‐tricalcium phosphate. With a high SR of 420%, the hydrogel manifested 93% of tetracycline release after 5 days, which qualified this guided bone regeneration material as an antibacterial drug delivery system for treating chronic periodontitis.^[^ ^111^ ^]^ To achieve therapeutic effects, several researchers have made remarkable progress in injectable hydrogel delivery systems. For delivering bovine serum albumin (BSA), Ma et al. developed injectable HSHs via in situ formation of Schiff base bonds between aldehyde HA and hydrazide‐modified γ‐PGA. The highest SR of the hydrogels could reach 70.^[^ ^112^ ^]^ In turn, Torchio et al. fabricated a series of injectable HSHs with marked self‐healing properties based on host‐guest interactions. High concentration (80 µg mL^−1^) of curcumin could be entrapped into the hydrogels and release progressively up to 4 days.^[^ ^113^ ^]^ Another injectable and high swelling multicomponent biomimetic gel (MBG) was developed by intercross‐linking polyamidoamine dendrimer G3, mesoporous silica NPs (MSNPs), and dendrimer‐templated Ag NPs with PEGDGE via the amine‐epoxy click reaction. PEG network delivered hydrophilic antibiotic isoniazid, whereas MSNs carried the hydrophobic antibiotic rifampicin. It was found that isoniazid could be quicky released while rifampicin could be released in a more sustained profile. Taken all the above facts, the MBGs with structural features for cartilage defect grafts could also offer antibiotic treatment benefits.^[^ ^114^ ^]^

To acquire controlled and targeted delivery of therapeutic molecules, there is a growing need to attain hydrogels whose swelling behaviors will change in reaction to different endogenous stimulus, for instance, pH,^[^ ^115^ ^]^ temperature,^[^ ^116^ ^]^ enzyme,^[^ ^117^ ^]^ redox conditions,^[^ ^118^ ^]^ or hypoxia.^[^ ^119^ ^]^ By far, the most widely used stimuli‐responsive hydrogels are pH‐sensitive hydrogels.^[^ ^120^ ^]^ These types hydrogels usually include at least one kind of polyelectrolyte within the network. With the variation in pH significantly influencing the molecular interactions like hydrogen bonding and electrostatic forces in the hydrogels,^[^ ^121^ ^]^ these “smart” or “intelligent” hydrogels will swell and deswell at specific pH, whereby prolong the protection of drugs in stomach or intestine, and dictate the release process.

As the only cationic natural polymers existing in nature,^[^ ^122^ ^]^ CS can provide a pH‐responsive controlled release profile of drugs and other bioactive molecules, resulting from the pH‐sensitive ionization of ‐NH_2_ groups in CS backbone.^[^ ^123^ ^]^ At acidic pH, the ‐NH_2_ groups will convert into ammonium groups (NH_3_^+^), which gives rise to an increase in the intermolecular electrostatic repulsion.^[^ ^124^ ^]^ While at basic pH, the NH_3_^+^ groups are significantly deprotonated, diminishing the charge imbalance.^[^ ^125^ ^]^ As such, CS‐based hydrogels will exhibit distinctive swelling behaviors in gastrointestinal tract, which is reliant on the tradeoff between the repulsive forces of polymer chains and the constraints imposed by cross‐linked network structure. And this feature may make them appropriate for being selected as targeted drug delivery systems (DDS).

**Figure 5:**
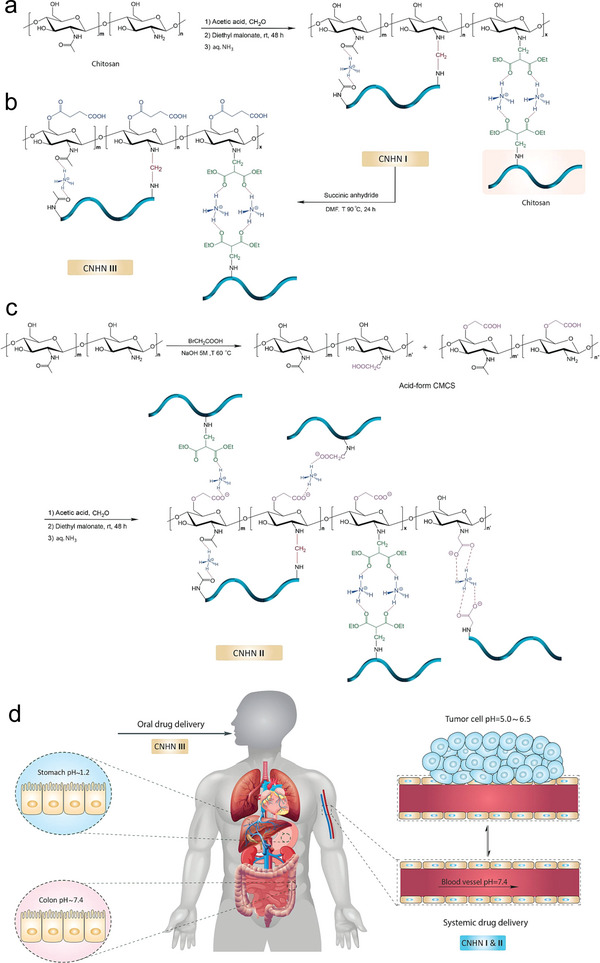
pH‐responsive HSHs for systemic and oral drug delivery. Synthesis pathways of CS nanohydrogel networks: a) CNHN I, b) CNHN III, c) CNHN II. d) Classification of hydrogels for oral and systemic drug delivery. Adapted with permission.^[^ ^129^ ^]^ Copyright 2022, Elsevier Ltd.

For this purpose, Rasool et al. attained pH‐sensitive hydrogels with antibacterial activity and biodegradability, by mixing CS with poly(*N*‐vinyl‐2‐pyrrolidone) (PVP) and using neutralized PAA as cross‐linker. The hydrogel samples manifested low swelling at acidic pH and high swelling at neutral pH. Their release profile of Ag‐sulfadiazine (antibiotic for burnt wounds) was in a controlled manner, which exhibited 91.2% of drug release within 1 h in PBS.^[^ ^123^ ^]^ Cesco et al. obtained physically crosslinked CS/PC blend hydrogel (CPB) and CS/DNA blend hydrogels (CDB) for releasing antineoplastic agent methylene blue (MB). It was determined that both hydrogels exhibited higher SRs in simulated gastric fluid (SGF). As such, in SGF (pH 1.2) at 25 °C, the cumulative release of MB respectively reached values of 55% and 96% for CPB and CDB after 6 h, whereas in simulated intestinal fluid (SIF) (pH 6.8), these values respectively decreased by 20% and 50%. This was due to the lower electrostatic interaction between the polymer and MB in CDB.^[^ ^126^ ^]^ Interestingly, a library of self‐healing hydrogels was fabricated based on Diels–Alder reaction between furan‐modified PC and maleimide‐modified CS. The click reaction substantially increased the cross‐linking density of PC/CS hydrogels, and did not cause any cytotoxicity. What's more, it brought significant transformation in the SR in response to temperature. Finally, the hybrid hydrogel indicated high encapsulation efficiency (53.67–65.27%) of 5‐fluorouracil, and sustained release performance under different simulated media, with the accumulative release retarded at pH 1.2 but increased at 7.4.^[^ ^127^ ^]^ Furthermore, Hafeez et al. developed injectable pH‐sensitive hydrogels by utilizing low molecular weight γ‐irradiated CS with higher hydrophilia, and varying concentrations of glycerol crosslinkers. Being able to inflow into the bacteria cell and rupture its metabolism more efficiently, γ‐irradiated CS‐based hydrogels displayed improved antibacterial activity. And the response against pH trigger made the hydrogel loaded with Montelukast sodium released all the drugs in 0.5 h in SGF. While in SIF, the drug was released in a more sustained manner (99.62% in 3 h). This evaluation results suggested the hydrogel as an injectable drug administration.^[^ ^128^ ^]^ Another group exploited γ‐irradiation to initiate the graft copolymerization between CS and l‐glutamic acid monomers. Ultimately, they attained highly porous, pH‐responsive, biocompatible, and biodegradable hydrogel beads. The highest SR (426%) and drug release (81.33% in 144 h) of a model anti‐tumor drug doxorubicin (DOX) was observed at pH 5.8, close to the pH of tumor (pH 5.5–6.0). And a significant cancer cell toxicity (78.18%) further qualified the CS‐based hydrogel beads as controlled carrier and deliverer of antitumor drugs for local cancer therapies.^[^ ^125^ ^]^ Interestingly, Omrani et al. reported three different CS nanohydrogel networks (CNHN I‐III) (**Figure** **5**a–c). The CNHN I and II had high SR in acidic environments, which were able to achieve an extraordinary loading efficiency of DOX (up to 95%). Dissimilarly, the CNHN III had substantial resistance in pH 1.2 but high swelling in pH 7.4, thus exhibited a high drug loading capacity of 5‐fluorouracil (up to 92%). Therefore, the CNHN I and II had been advocated for systemic drug delivery, while the CNHN III was nominated for oral drug delivery (Figure 5d).^[^ ^129^ ^]^

Analogously, the protonation and deprotonation at specific pH of ‐COOH groups of anionic natural and synthetic polymers,^[^ ^130^ ^]^ for example, SA, CMC, or PAA, have been exploited to construct pH‐responsive hydrogel DDS as well. Apoorva et al. prepared a SA‐Gum tragacanth composite hydrogels, which revealed higher swelling behavior, loading efficiency, and more sustained release of naturally derived antioxidant phenolic compounds in SIF.^[^ ^131^ ^]^ Based on AA/xanthan gum biopolymer, Hajikhani et al. synthesized semi‐IPNs showing the highest and lowest SRs in basic and acidic environments, respectively.^[^ ^132^ ^]^ Suhail et al. and Nath et al. exploited the anionic property of AA as well. The former team synthesized Carbopol934‐g‐PAA hydrogels by free‐radical polymerization,^[^ ^133^ ^]^ while the latter one utilized CMC‐g‐PAA and layered double hydroxide.^[^ ^134^ ^]^ With pH‐responsive swelling behaviors same as the abovementioned semi‐IPNs, both the hydrogels demonstrated a greater drug release at basic (pH 7.4) mediums. Notably, a pH‐responsive biocompatible hydrogel was prepared by grafting HEMA onto soy protein isolate. The hydrogel exhibited faster swelling rate and higher cumulative drug release (70%) at pH 7.4, which was decreased to 30% at pH 1.2.^[^ ^135^ ^]^ Therefore, these hydrogels are ideal candidates for intestinal targeted drug delivery. In another study, a combination of PAM, NaCMC and MgO NPs yielded smart hydrogels with appropriate drug release conditions being at 37.5 °C and pH 4.1.^[^ ^136^ ^]^

**Figure 6:**
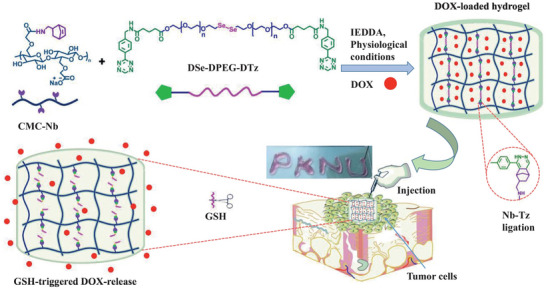
Redox‐responsive HSHs loaded with DOX for cancer therapy. Schematic representation for the construction and drug release mechanism of the redox‐responsive hydrogels. Adapted with permission.^[^ ^12f^ ^]^ Copyright 2022, Elsevier B.V.

HSHs sensitive to other stimuli have also emerged for targeted drug delivery. For example, redox‐responsive hydrogels can release the payload of therapeutic agents when met diseased tissues (e.g., tumor) with over expressed levels of reducing agent glutathione (GSH).^[^ ^137^ ^]^ An example for the use of reduction‐responsive HSHs was developed by Lim's team via inverse electron demand Diels‐Alder reaction (IEDDA). The hydrogels were formulated from alginate‐norbornene (Nb) and PEG‐tetrazine (Tz) based disulfide cross‐linker. It possessed high SRs, injectability, cytocompatibility, and remarkable DOX loading efficiency (92%). Moreover, after 11 days, the DOX release rate of the hydrogels in 10 mm GSH solution (93%) was much higher than in PBS (36%).^[^ ^138^ ^]^ In the next year, the same team replaced the alginate in the previous click hydrogels by CMC, and used another diselenide‐based cross‐linker bearing two terminal Tz groups (DSe‐DPEG‐DTz) (**Figure** **6**). The resulting hydrogels also exhibited excellent DOX loading efficiencies (>85%), and faster DOX release rate (99%, after 12 h) in 10 mm GSH solution.^[^ ^12f^ ^]^ Therefore, this preparation strategy offered a general paradigm for the progress of redox‐responsive drug release applications.

Despite of the high drug loading efficiency and sustained release behavior of above HSHs, the biological studies carried out have been confined to only in vitro assays. Undoubtedly, it is insufficient to confirm that these hydrogels are suitable for treating specific human diseases. Therefore, in the future, there is an urgent need to roundly study the in vivo safety and therapeutic effect of swelling‐controlled DDS for practical implementation.

## 3 Non‐Swelling Hydrogels

Although hydrogels will not disintegrate in water due to its physically and/or chemically crosslinked 3D network structure,^[^ ^1^, ^139^ ^]^ the swelling of HSHs may cause suboptimal performance and unpredicted outcomes after applied in vivo. For example, the mechanical performance and dimensional stability of HSHs will be drastically weakened.^[^ ^140^ ^]^ And in particular, swelling will induce compression on local neighboring organs or tissues, reduce the adhesion and cause detachment from the installation site.^[^ ^141^ ^]^ Hence, HSHs are unaccommodated for biomedical areas which need excellent integrated properties to withstand long‐period load, keep original sizes, and shapes, avoid irritations to adjacent fragile tissues, or maintain durable and strong bioadhesion, such as bioelectronics, human organ implants, scaffolds in nerve, blood vessels, and cartilage tissue engineering, bioadhesive sealants in internal surgical operations, etc.^[^ ^139^, ^142^ ^]^ In this case, non‐swelling hydrogels with high‐strength and versatile functionalities rise in response to the actual requirements in these areas.

Non‐swelling hydrogels (NSHs), also known as anti‐swelling, non‐swellable, or swelling‐resistant hydrogels, refer to a type of hydrogel whose swelling behavior is very nearly an equilibrium state.^[^ ^141^, ^143^ ^]^ In other words, these NSHs can retain their initial shapes and intrinsic properties underwater,^[^ ^139^ ^]^ Common strategies on developing NSHs can be categorized as increasing the crosslinking density,^[^ ^13a–d^ ^]^ controlling the polymer/water interaction parmater,^[^ ^13^, ^144^ ^]^ introducing thermosensitive polymers,^[^ ^145^ ^]^ and so on.^[^ ^143^, ^146^ ^]^ Obviously by virtue of different methods and therein distinct chemical designs to deliver non‐swelling features can bring up different functions to the hydrogels. Based on these reasons, we have summarized and classified the recent five years advanced researches on NSHs as follows (**Table** **3**).

**Table 3:** Brief summary of the constructions, crosslinking mechanisms, properties, and biomedical applications of NSHs

Constructions	Hydrogel systems	Crosslinking mechanisms	Properties	Biomedical applications	Ref.
By increasing crosslinking density	HA‐norbornene, HA‐ methylphenyltetrazine	IEDDA reaction	Non‐swelling, transparent, cytocompatible biodegradable	Not‐involved	[13b]
	HB‐PEGDA, HA‐SH, colloidal photonic crystal	Michael addition reaction	Non‐swelling, robust, photonic, biocompatible	Bio‐optical application	[157]
	CS, PAM, antibiotics	Double covalent network	Anti‐swelling, robust, adhesive, antibacterial	Intraoral wound healing	[219]
	PVA, TA	Multiple H‐ bonding	Hierarchically anisotropic, super‐strong, nonswellable, cytocompatible	Not specific	[13d]
	PVA, DMSO	H‐bonding	Anti‐swelling, strong, wet adhesive	Not specific	[13c]
	PEGDA, TA	H‐bonding	Non‐swelling, tough, notch insensitive, self‐healing, tailorable topography, wet adhesion, on‐demand detachment, biocompatible	Underwater wound sealant	[227]
	P(AA‐*co*‐LMA), CTAB	Hydrophobic association, electrostatic interaction, H‐bonding	Tough, self‐recovery, anti‐swelling, conductive, wet adhesive, strain‐sensitive	Underwater sensor	[180]
	SA, P(AM‐*co*‐AA), Fe^3+^	Dual ionic crosslinking	Robust, tough, fatigue‐resistant, self‐recovery, pH‐triggered healing, shape memory, anti‐swelling, 3D‐printable	Not specific	[183]
	P(AA‐*co*‐DMAA), Al(OH)_3_	Metal nanoparticle coordination	Nonswellable, ultra‐strong, superoleophobic	Not involved	[13a]
	PSBVI, graphene	Cation‐π interaction	Conductive, bioadhesive, non‐swelling, high conformability, biocompatible	Bioelectronics	[243a]
	GelMA, acryloyl‐β‐CD, β‐CD‐rGO	Covalent bonding, host‐guest complexation	Conductive, non‐swelling, biocompatible, photothermal antibacterial, osteogenic	Bone defect repair	[231]
	‐CHO‐capped tetra‐arm PEG, ‐ONH_2_‐capped PEG‐UPy‐PEG	Chemical and micelle physical dual crosslinking	Injectable, strong, tough, stable, non‐swelling, an‐fatigue, tissue‐adhesive, cytocompatible	Not specific	[195b]
	pHEMA, alginate, Fe^3+^	Interpenetrating covalently and ionically crosslinked networks	Non‐swelling, stiff, tough, cytocompatible	Not involved	[203]
	Cellulose, PAM, aniline	H‐bonding, covalent bonding	Conductive, tough, stretchable, elastic, non‐swelling, biocompatible, underwater strain‐sensitive	Strain sensor	[47]
By regulating polymer‐water interaction	P(AA‐*co*‐MEA), nucleobase pairs, DMSO‐water binary solvent	H‐bonding	Non‐swelling, anti‐freezing, wet adhesive, tough, mechanically stable	Sweaty skin sensor	[144]
	P(AA‐*co*‐MEA), CMC, Al^3+^, DMSO‐water binary solvent	Covalent bonding, ionic crosslinking, H‐bonding	Anti‐swelling, anti‐freezing, moisturizing retention, underwater/oil strain‐sensitive, optically adjustable, flexible	Multipurpose biosensor	[13f]
	Hydrogel components: P(HEA‐*co*‐DMA), clay nanosheets, Organogel components: PBA	Covalent bonding	Stretchable, tough, enduring anti‐swelling, low dehydration	Not specific	[206b]
	Hydrogel layer: P(AA‐*co*‐HEA)/MXene, Lipogel layer: hydrophobic lipid gel	Covalent bonding	Anti‐swelling, anti‐hydration, long‐term ultra‐stable, underwater mechanosensitive	Underwater mechanosensing	[210b]
By including thermo‐responsive polymer	2‐arm thiol‐ terminated Pluronic l‐64 PEG, 3‐arm alkyne terminated PEG	Thiol‐yne click reaction	Non‐swelling, cytocompatible	Soft tissue scaffold	[145a]
	Di‐acrylated Pluronic F127	Covalent bonding	Non‐swelling, autoclavable, degradation‐resistant	Not specific	[145b]
	HA‐MA, Pluronic F127‐bis‐acryloyloxy acetate	Covalent bonding	Anti‐swelling, injectable, wet adhesion, biocompatible	Hemostasis and wound sealing	[13h]

### 3.1 Achieved by Increasing Crosslinking Density

Densifying crosslinking density is the most crucial method to construct hydrogels with anti‐swelling ability. This is because the increased structural tightness not only limits intake of water molecules whereby restricts swelling, but also brings better mechanical performance.^[^ ^147^ ^]^ To this end, recent attempts have been made to select various types crosslinking methods to yield NSHs. From the perspective of chemistry, these methods can be categorized into utilizing chemical cross‐linking or physical cross‐linking or a combination of both. The huge differences in the binding energy and reversibility between the two cross‐linking support the tailoring of distinct desired properties, such as robustness, ductility, injectability, or self‐healing ability. Hence, in this section, various NSHs was presented on account of crosslinking patterns.

#### 3.1.1 Covalent Crosslinking

Chemical cross‐linking techniques can produce hydrogels with high crosslinking density,^[^ ^148^ ^]^ and more controlled physicochemical properties,^[^ ^149^ ^]^ thus have been adopted in the preparation of NSHs. In general, by virtue of externally initiated free radical polymerization and chemical crosslinking reagents, which involves formation of strong and permanent covalent bonds that interconnect polymers, can achieve hydrogels with good stability under physiological conditions.^[^ ^150^ ^]^ However, these hydrogels tend to be brittle and not injectable, because the energy dissipation of covalent bonds under stress will cause unrecoverable rupture.^[^ ^151^ ^]^ Besides, the addition of initiators and other reactive chemicals may increase the cytotoxicity of the hydrogels, which impedes their biomedical application in future.^[^ ^152^ ^]^ Therefore, stable covalent bonds are often combined with dynamic noncovalent interactions for the design of practical non‐swelling hydrogel systems (Section 3.1.3). And the biocompatibility of the hydrogels is mainly improved by introducing biopolymers therein.

**Figure 7:**
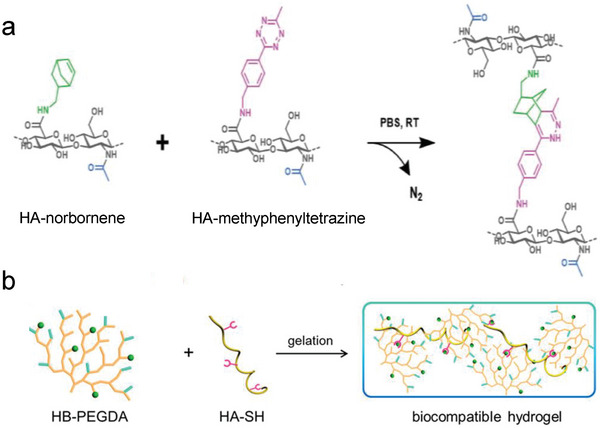
Synthesis of NSHs via dynamic covalent crosslinking. a) Schematic illustration of HA‐based NSHs formed via IEDDA reaction between HA‐norbornene and HA‐methylphenyltetrazine. Adapted with permission.^[^ ^13b^ ^]^ Copyright 2020, Wiley‐VCH Verlag GmbH & Co. KGaA. b) Preparation of NSHs via thiol‐ene Michael addition between HB‐PEGDA and HA‐SH. Adapted with permission.^[^ ^157^ ^]^ Copyright 2018, American Chemical Society.

Recently, dynamic covalent bonds have emerged for addressing the issues in fabricating NSHs solely by chemical cross‐linking. It can reversibly convert polymer precursors into a gel, which is beneficial for conferring NSHs with multiple function. In this area, the most employed are click reactions, including thiol‐yne/ene addition,^[^ ^145^, ^153^ ^]^ Michael addition,^[^ ^154^ ^]^ IEDDA,^[^ ^13b^ ^]^ or a combination thereof.^[^ ^155^ ^]^ In click chemistry, high yields of hydrogel products can be achieved by mixing two components equipped with functional groups at mild condition.^[^ ^156^ ^]^ For example, Delplace et al.^[^ ^13b^ ^]^ first developed two modified HA precursors, that is, HA‐norbornene and HA‐methylphenyltetrazine (**Figure** **7**a). After mixing, gelation was caused through IEDDA reaction between the two components within tunable times. By optimizing HA molar mass, transparency and non‐swelling property were achieved with very low polymer content (0.5% (w/v)). The IEDDA hydrogels exhibited biodegradability and cytocompatibility as well, which enabled them pertinent for cell culture and retinal explant imaging. More advantageously, the hydrogels doubled the multiphoton imaging time (>38 h) of embedded retinal explants of the existing standard agarose thermo‐gels (<20 h). In the works of Macdougall et al.,^[^ ^145a^ ^]^ two straightforward routes were put forward to render the thiol‐yne PEG hydrogel non‐swellable. One route increased the cross‐linking density by using multi‐arm PEG precursors instead of previously reported 2‐arm PEG.^[^ ^153^ ^]^ The other introduced a thermo‐sensitive segment into the PEG network, which would be elaborately descripted in Section 3.3. The multi‐arm click‐hydrogels showed shorter gelation time than the thermo‐responsive hydrogel. And both pathways resulted in preserved robust mechanical properties under physiological conditions over time. Ulteriorly, as shown in Figure 7b, hyperbranched PEG diacrylate (HB‐PEGDA) polymers with high amount of pendant vinyl groups were synthesized to quickly form numerous junctions with thiolated HA (HA‐SH) via Michael addition reaction. Rooting in its relatively compacted network structure, this hydrogel showed non‐swelling characteristics, thus could maintain robust structural integrity under diverse complex physiological conditions.^[^ ^157^ ^]^

In addition to click reactions, addition‐fragmentation chain transfer (AFCT) reaction has regeneration nature as well.^[^ ^158^ ^]^ Therefore, Wu et al. synthesized HA‐SH and allyl sulfide‐functionalized ε‐poly‐l‐lysine to create a non‐swellable and biocompatible hydrogel system, based on reversible AFCT reaction between thiol and allyl sulfide functional groups under UV irradiation. The modulus of the hydrogel was reversed and could be tuned by controlling UV irradiation dosage, and the patterning of fluorescent markers was dynamic as well, which facilitated the imitation of natural ECM. Thus, this dynamically controllable hydrogel displayed better advantages for tissue engineering applications.^[^ ^159^ ^]^

In summary, the above two dynamic chemical cross‐linking strategies both occupy an important position in preparing NSHs constituted by a single covalent network structure. But differently, in click chemistry, the mechanics and biochemistry of hydrogels can be regulated through varying the concentration or architecture of the polymers, while in AFCT reaction, the turning can be dynamically and reversibly realized through controlling the dose of required external stimulus, for example, light irradiation.

#### 3.1.2 Noncovalent Crosslinking

Physical crosslinking approach have drawn much attention in manufacturing NSHs due to the merits of noncovalent bonds in reversibility and configurability. Physical hydrogels can be formed via varied noncovalent interactions (e.g., hydrogen bonding, ionic complexing/electrostatic interaction, hydrophobic association, or metal coordination).^[^ ^13^, ^160^ ^]^ Compared to covalent bonds, intra‐ and intermolecular noncovalent interactions are quite weak, and may demonstrate sensitivity to certain external stimuli such as pH, temperature or ionic strength.^[^ ^161^ ^]^ Therefore, noncovalent interactions have frequently come in combination (i.e., multiple noncovalent interactions). Meanwhile, by the presence of multiple hydrogen bonds or hydrophobic association, the molecular chain may self‐assembly into ordered microdomains or nanodomains, which further intensify the physical crosslinking.^[^ ^162^ ^]^ Therefore, the multiple and dense physical crosslinking sites can endow hydrogels with excellent anti‐swelling ability. What's more, the “weaker” crosslinks that are readily reversible can effectively dissipate energy under external stress, while the “stronger” one can provide a strong mechanical support. Thus, the physical crosslinked NSHs unite appealing functionalities like injectability, rapid self‐repairing, shape memory, recyclability, and extraordinary mechanical properties, which is advantageous for practical applications.

##### H‐bonding

Among a variety of noncovalent interactions, H‐bonding is most widely existent in physically crosslinked hydrogel systems. H‐bonding is the electrostatic attraction between two vicinal polar groups, each of which contains a H‐bonded electronegative atom and one termed as donor while the other as acceptor.^[^ ^161^, ^162^ ^]^ Single H‐bond has low binding energy and strong competition with water, thus plays a minor role in stabilizing the hydrogel network in water.^[^ ^163^ ^]^ But multiple H‐bonds can be as strong as covalent bonds,^[^ ^79^, ^164^ ^]^ and in a macromolecule, the self‐assembling structures of multiple H‐bonds such as crystallite can provide an additional stabilization.^[^ ^165^ ^]^ Therefore, multiple H‐bonds make a major contribution to prepare H‐bonding crosslinked NSHs with high strength and stability. And the dynamic nature of H‐bonding can endow the hydrogels with admirable rheological properties and injectability as well.

**Figure 8:**
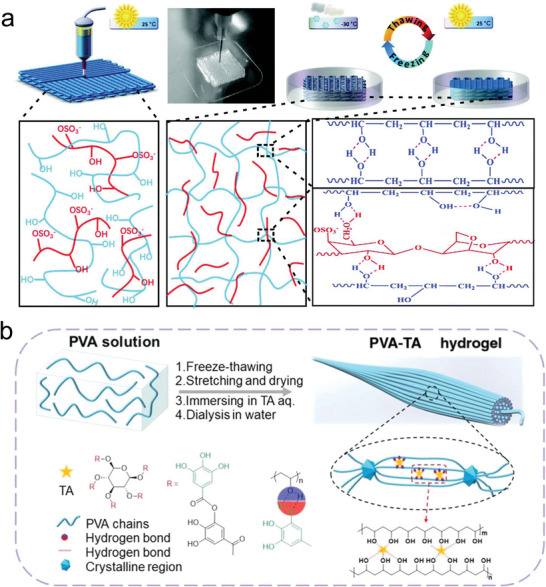
Synthesis of NSHs via H‐bonding crosslinking. a) Illustrations for the preparation and post‐processing of the 3D printed hydrogels composed by PVA and κ‐carrageenan; Adapted with permission.^[^ ^169^ ^]^ Copyright 2019, The Royal Society of Chemistry. b) Fabrication illustration of the tendon‐inspired PVA‐TA hydrogel. Adapted with permission.^[^ ^13d^ ^]^ Copyright 2022, American Chemical Society.

**Figure 9:**
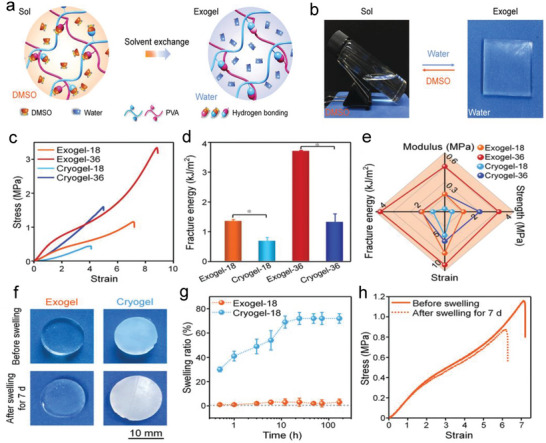
Synthesis and characterization of NSHs based on solvent exchange‐intensified H‐bonding crosslinking. a) Fabrication of stiff and non‐swelling PVA exogel. b) Reversible sol‐gel transitions of PVA exogel achieved by altering solvent. c) Tensile stress‐strain curves, d) fracture energy, and e) comprehensive mechanical properties of PVA exogel and cryogel with varied PVA content. f) Optical images and g) SRs of PVA exogel and cryogel in water at determined time point. h) Tensile stress‐strain curves of PVA exogel before and after swelling for 7 days. Adapted with permission.^[^ ^13c^ ^]^ Copyright 2020, Wiley‐VCH GmbH.

The most classic non‐swelling hydrogel relying on H‐bonding is PVA hydrogels. PVA chains can be crosslinked by the crystallization in PVA during repeated freeze‐thawing (FT) process (cryogel), which was induced by the H‐bonding between the pendant ‐OH groups.^[^ ^166^ ^]^ In addition, the abundant ─OH groups offer many opportunities for PVA forming H‐bonding with other reactive substances.^[^ ^167^ ^]^ It has been reported that traditional frozen‐thawed PVA hydrogels are compliant and brittle.^[^ ^168^ ^]^ In order to prepare PVA‐based NSHs possessing higher stiffness and toughness, attention have been paid to synchronously take advantage of the two H‐bonding crosslinking methods of PVA. For instance, Jiang et al. first prepared 3D printed hydrogel inks with outstanding rheological properties based on wide‐ranging H‐bonding and van der Waals interactions between PVA with κ‐carrageenan (**Figure** **8**a). With the aid of followed FT processes, the mechanical and anti‐swelling properties of the printed structures were strengthened.^[^ ^169^ ^]^ Notably, soaking in sodium silicate solution after FT could convert PVA into layered hydrogels with a compact outside and a porous center. Crosslinked by ordered polarized H‐bonds, the cover layer endowed the hydrogels superior robustness against swelling. And the hydrogels could retain exceptional mechanical stability in harsh liquid conditions like strong acidic/alkaline, concentrated electrolytes, and salting‐in and H‐bond‐breaking reagents even at elevated temperatures.^[^ ^170^ ^]^ Luo's team have also achieved the goal of toughening PVA‐based NSHs through employing different post‐treatments after FT. As the first example, the subsequent step of immerging PVA/agarose precursor in (NH_4_)_2_SO_4_ aqueous solution induced phase separation micro‐regions thereby increased crystallinity of PVA. The obtained PVA/agarose hydrogels exhibited high tensile strength of 1.1 MPa, tensile strain of 324% and compressive stress of 12.5 MPa.^[^ ^171^ ^]^ Later, exploiting a pre‐stretching strategy and an energy dissipation mechanism offered by H‐bonding between PVA and tannic acid (TA), they fabricated a super‐strong PVA/TA hydrogel (Figure 8b). Compared to previously reported swelling‐resistant PVA/TA hydrogel,^[^ ^172^ ^]^ this strategy induced hierarchically anisotropic structures similar to tendon. Consequently, the acquired hydrogel showed a huge breakthrough in tensile stress (19.3 MPa) and toughness (32.1 MJ m^−3^).^[^ ^13d^ ^]^ Both the two PVA‐based hydrogels barely expanded in deionized water and PBS, thus could maintain their initial strength after water immersion for a week. And the bio‐inspired PVA/TA hydrogel have further been investigated to be non‐swelling in simulated body fluid solutions. Benefiting from the biocompatibility of starting materials and dialysis in water to remove residues, the two hydrogels displayed splendid cell compatibility, promoting its potential as load‐bearing devices. Particularly meaningful is that Xu et al. proposed a facile but highly effective solvent exchange approach to substitute the complicate cyclic FT process and further strengthen the PVA hydrogels (**Figure** **9**a,b). Key to this strategy was first choosing a good solvent (i.e., DMSO) to dissolve PVA, which favored a fully stretched polymer conformation of PVA without any nano‐aggregated states. Subsequently, the switch of DMSO into a poor solvent (i.e., water) intensified the intermolecular H‐bonding of PVA and resulted the formation of cross‐linked hydrogel networks (named as exogels). Due to the different polymer conformation and aggregation state in the two preparation routes, the exogels exhibited better stiffness, toughness, transparency, and anti‐swelling performances than cryogels though the chemical compositions were exactly the same (Figure 9c–h).^[^ ^13c^ ^]^

Some natural polymers like agarose,^[^ ^173^ ^]^ and cellulose,^[^ ^174^ ^]^ have also been utilized to prepare NSHs. Thanks to existed numerous intermolecular and intramolecular hydrogen bonds, its molecular chains are prone to aggregate to form Table 3D hydrogel network which hardly swell in water.^[^ ^175^ ^]^ Taking advantage of the stiff helical chains of agarose, which was driven by cooling hot agarose solution below the critical gelation temperature, Topuz et al. successfully synthesized a kind of nanocomposite non‐swelling agarose hydrogels. The incorporation of bioactive nanosilicates (Laponite) enhanced the biological activity of bioinert agarose matrices without affecting its non‐swelling nature.^[^ ^176^ ^]^ Interestingly, by means of surface modification of cationic CS onto supramolecular fiber structure of oxidized dialdehyde bacterial cellulose (DABC), Pedige et al. invented hydrogel composites integrating pH‐responsive stiffness and pH‐insensitive swelling resistance. In order to prevent CS from diffusing out of the composites, the first formed imine bonds between CS and DABC which was unstable in acid conditions, were then reduced into secondary amines.^[^ ^177^ ^]^

##### Hydrophobic Association

Increasing research attention has also been dedicated in deformable self‐assembly structures like micelles of amphiphilic macromolecules based on hydrophobic association. In comparison to assembling structures induced by multiple hydrogen bonds, that caused by hydrophobic aggregation are more flexible. Accordingly, the structures have been employed as reversible physical crosslinkers to efficiently dissipate energy under repetitive loading and provide substantial toughness or other specific properties to NSHs.^[^ ^178^ ^]^

**Figure 10:**
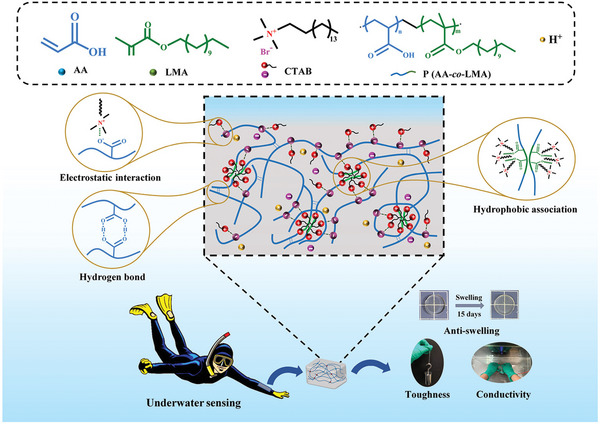
Synthesis of NSHs via hydrophobic association. Preparation scheme of the tough, anti‐swelling, and conductive P(AA‐*co*‐LMA)_CTAB_ hydrogel. Adapted with permission.^[^ ^180^ ^]^ Copyright 2022, American Chemical Society.

For instance, based on the rigid self‐assembled tri‐helix structure of gelatin and reversible aggregation of free and gelatin‐grafted hydrophobic moieties, Feng et al. prepared non‐swelling and thermoplastic supramolecular gelatin hydrogels, which become highly malleable upon heating but extremely stretchable and tough after cooling to room temperature.^[^ ^179^ ^]^ By copolymerizing hydrophilic AA and hydrophobic LMA in the presence of cationic surfactant cetyltrimethylammonium bromide (CTAB), another supramolecular hydrogel was facilely synthesized via the formation of hydrophobic association zones (**Figure** **10**). By forming electrostatic interaction with COO^−^ on AA segments, CTAB could further promote the toughness and hydrophobicity of the hydrogel network. The resulting hydrogel showed prominent swelling resistance, superior tensile strength and stretchability, rapid room‐temperature self‐recovery, and stable conductivity in wet environments. Notably, anhydrous ethanol treatment would partially destruct the hydrophobic association zones and improve the motion of polymer chains, which facilitated the diffusion of polymer chains for forming strong adhesion with substrates via diverse interactions.^[^ ^180^ ^]^ Moreover, through decreasing the hydrophobic domains’ size and interacting with charged solid surfaces of many materials, the introduction of electrostatic interactions can also promote the adhesion of hydrophobic hydrogels.^[^ ^181^ ^]^

##### Ionic Complexing/Electrostatic Interaction

Leveraging ionic complexing/electrostatic interaction as physical crosslinks is a facile and alternative path to construct NSHs with good electrical properties. Ionic cross‐linking junctions formed by strong coordination bonds between anionic groups of polymers and multivalent metal cations (Fe^3+^, Al^3+^, Ca^2+^, Mg^2+^, La^3+^),^[^ ^182^ ^]^ can be easily achieved by incubating as‐prepared hydrogel precursors in metal ions aqueous solution. The addition of metal cations can not only impart hydrogel anti‐swelling properties, but also sensitive conductivity, providing the hydrogel a promising application as wearable sensors. And the effortless tuning of ions concentration eases the modulation of cross‐linking density.

**Figure 11:**
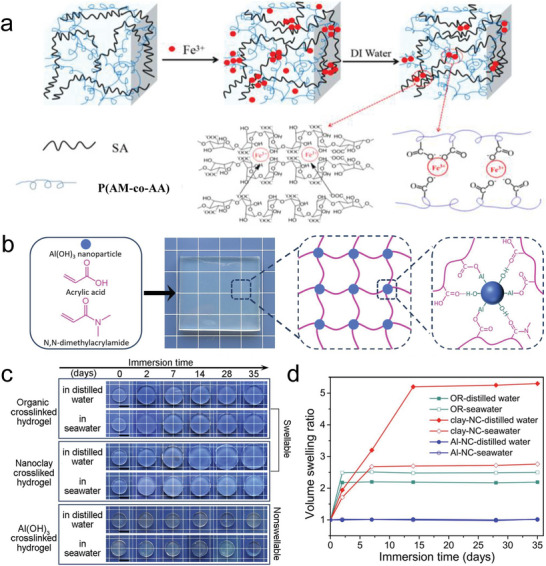
Synthesis and characterization of NSHs based on ionic crosslinking and metal nanoparticle coordination, respectively. a) Fabrication scheme of dual ionically cross‐linked DN SA/P(AM‐*co*‐AA)/Fe^3+^ hydrogels. Adapted with permission.^[^ ^183^ ^]^ Copyright 2018, American Chemical Society. b) Synthesis illustration of Al(OH)_3_ crosslinked hydrogel (Al‐NC gel). c) Photographs and d) volume SRs of organic crosslinked hydrogel (OR gel), nanoclay crosslinked hydrogel (clay‐NC gel), and Al‐NC gel immersed in distilled water or seawater (scale bar: 10 mm). Adapted with permission.^[^ ^13a^ ^]^ Copyright 2019, Elsevier B.V.

Accordingly, a multifunctional SA/P(AM‐*co*‐AA)/Fe^3+^ non‐swelling hydrogel with robustness and toughness were manufactured via a dual Fe^3+^‐COO^−^ cross‐linking approach (**Figure** **11**a). Profiting from the reversible nature of ionic cross‐linking, the hydrogels also exhibited high fatigue resistance, self‐recovery, pH‐triggered healing capacity, shape memory, and reversible gel‐sol transition favored by pH regulation.^[^ ^183^ ^]^ The introduction of Fe^3+^ into the P(AM‐*co*‐AA)‐CS hydrogel network also yielded a hydrogel with eminent mechanical strength, self‐recovery, and anti‐swelling behavior.^[^ ^184^ ^]^ Based on the synergistic contribution of ionic coordination, electrostatic interaction and H‐bonding, Wu et al. prepared a QCS/PAA/Fe^3+^ hydrogel system with outstanding anti‐swelling, mechanical and self‐healing properties. QCS also endowed the hydrogel excellent adhesiveness, while the existence of dynamic ions (Fe^3+^, Cl^−^) provided surprising conductivity.^[^ ^185^ ^]^ Through adding ionic liquid and Al^3+^ into starch/PVA DN structure, Lu et al. developed an anti‐swelling conductive hydrogel with high stretchability, tensile strength, and good anti‐freezing properties.^[^ ^186^ ^]^ In addition, based on the chelation between multi‐ionic biomineral (containing Mg^2+^, Ca^2+^, CO_3_^2−^, and PO_4_^3−^) and the matrix of biocompatible PVA and SA, another ionic crosslinked conductive NSHs was constructed. Meaningfully, the presence of Mg^2+^ and PO_4_^3−^ prohibited the crystallization in the biomineral, leading to stable network with excellent mechanical performance and anti‐freezing property.^[^ ^187^ ^]^

Electrostatic interaction can be the driving force for gelification as well. In comparison to ionic complexing, the formation of electrostatic interaction not strictly require the presence of ions. It can appear between various substances equipped with opposite charges, for example, polyelectrolytes,^[^ ^188^ ^]^ polyelectrolyte and other inorganic materials,^[^ ^189^ ^]^ or ionic monomers,^[^ ^190^ ^]^ and so forth. Interestingly, Sun et al. employed the stable electrostatic interactions between zwitterion poly(sulfobetaine methacrylate) (PSBMA) chains to deliver hydrogel with anti‐swelling property. The physically crosslinked PSBMA hydrogel have fast autonomous healing capacity, conducive to regain properties and prolong service life of hydrogel for underwater sensing.^[^ ^191^ ^]^

##### Metal Nanoparticle Coordination

Metal nanoparticles are a type of distinguished inorganic cross‐linking agents on account of their capacity to form strong coordination interactions with polymer matrix. In addition, it is very favorable for incorporating nanoparticles to reinforce the network structure, as the enhancement mechanism resembles that of natural animal and plant tissue.^[^ ^192^ ^]^ Therefore, several works have exploited metal nanoparticles to enhance the anti‐swelling property and mechanical strength of hydrogels, through copolymerization of two monomers in metal nanoparticles solution.

To exemplify, employing the topotactic chelation between Al(OH)_3_ and ─COOH groups on polymer chains, an ultra‐strong nanocomposite non‐swelling hydrogel was prepared via photopolymerization of AA and *N,N*‐dimethylacrylamide monomers in Al(OH)_3_ solution (Figure 11b–d). This hydrogel not only demonstrated volume stability over an extended period of time, but also exceptional mechanical performances (tensile strength, elastic modulus, and compressive strength respectively up to 9.2, 52.3, and 48.5 MPa).^[^ ^13a^ ^]^ Adopting the same principle of crosslinking, Xu et al. synthesized a similar high‐strength nanocomposite NSHs, with *N,N*‐dimethylacrylamide and Al(OH)_3_ in the abovementioned hydrogel system respectively replaced by *N*‐vinyl‐2‐pyrrolidone and Al_2_O_3_. Specially, the strong H‐bonding existed in the water‐glycerol bi‐solvent solutions imparted the prepared hydrogels excellent anti‐dehydration capacity.^[^ ^193^ ^]^ Advantageously, the physical properties of the both nanocomposite hydrogels could be simply optimized by altering the metal nanoparticle content as well as the monomer concentration.

#### 3.1.3 Hybrid Crosslinking

Combining chemical and physical cross‐linking (i.e., hybrid) is a simple and effective method to increase the number of crosslinking sites. Under load‐bearing conditions, the permanent chemical cross‐linking can stabilize the deformation and maintain the configuration of the hydrogel, while the reversible physical cross‐linking can dynamically break and recombine to release energy.^[^ ^194^ ^]^ So simultaneously utilizing the two types interactions can balance the trade‐off between strength and toughness of hydrogels. Besides, it can also conquer the defects of rapid degradation of single type cross‐linking.^[^ ^195^ ^]^ Therefore, employing hybrid crosslinking methods can achieve hydrogels with not only anti‐swelling ability, but also comprehensive mechanical performance and excellent stability under physiological environments. Chemically physically crosslinked hydrogels can be conceived as DC hydrogels when exploiting different genres of interactions to interconnect a single polymer,^[^ ^160^, ^172^, ^196^ ^]^ and/or DN hydrogels comprised of two cross‐linked networks with strong asymmetric properties.^[^ ^151^, ^197^ ^]^ Through regulating cross‐linking density of each interaction, NSHs with high mechanics can be acquired.

**Figure 12:**
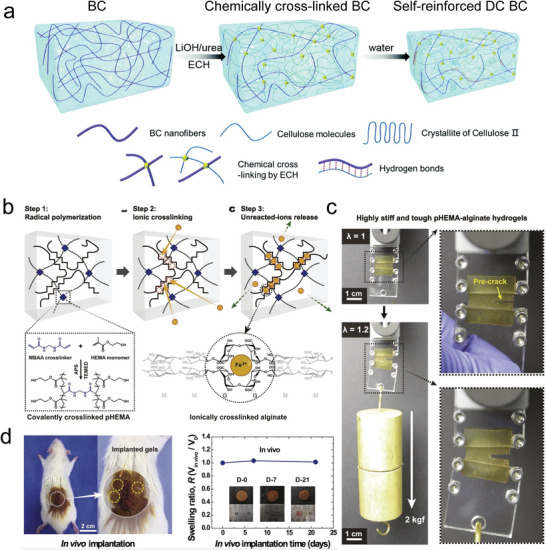
Synthesis and characterization of NSHs based on hybrid crosslinking. a) Schematic illustration of preparing self‐reinforced DC BC hydrogels. ECH, epichlorohydrin. Adapted with permission.^[^ ^198^ ^]^ Copyright 2019, The Royal Society of Chemistry. b) Reaction sequence of pHEMA‐alginate hydrogels. c) Demonstration of high stiffness and toughness and d) in vivo swelling ratios of pHEMA‐alginate hydrogels with hydrogel implanted in rats for 7 and 21 days. MBAA, *N*,*N*′‑methylenebisacrylamide. Adapted with permission.^[^ ^203^ ^]^ Copyright 2018, Elsevier B.V.

Through embedding a distinct nanofiber‐network‐self‐reinforced structure in the chemically cross‐linked cellulose framework containing different crystal forms, a new DC BC hydrogel had been invented (**Figure** **12**a). No swelling occurred for a good while because the remove of the alkali solvent in water leaded to a dense structure via H‐bonding.^[^ ^198^ ^]^ In addition, Lu et al. fabricated DC NSHs through covalently cross‐linking BSA via photoreaction‐induced dityrosine bonds and physically cross‐linking BSA by thermal denaturation. The gels demonstrated a high compressive strength (37.81 MPa) and tensile strength (0.62 MPa), as well as non‐swelling behaviors in deionized water whether at pH 3.0 or pH 10.0. Originating from the existed dynamic physical interactions, BSA DC gel also displayed large hysteresis, rapid self‐recovery, and eminent anti‐fatigue capacities.^[^ ^199^ ^]^ By virtue of introducing strong micelle cross‐linking into chemically crosslinked PEG network, two kinds injectable and tough DC hydrogels were developed by Qin et al.^[^ ^195^, ^200^ ^]^ Both kinds DC hydrogels exhibited enhanced mechanical properties and stability, which could maintain relative integrity of the hydrogel network under physiological conditions. Besides, these hydrogels also demonstrated remarkable fatigue resistance, bioadhesion, and cytocompatibility. Chen et al. prepared an antibacterial hybrid hydrogel with anti‐swelling property by introducing ionic liquids with hydrophobic alkyl chain into covalently crosslinked PAA network. Ionic liquids effectively increased the electrostatic and hydrophobic interactions in the hydrogel network, thus enabling the hydrogel resist the interference of water. Through tuning the length of alkyl chains in ionic liquids, the hydrogel received high tensile strength, and sensitivity, even in seawater.^[^ ^160b^ ^]^ Based on heat‐induced micellar copolymerization of AA, octadecyl methacrylate, and SBMA, Xu et al. also successfully fabricated multiple crosslinked hybrid NSHs. *N,N*′‐methylenebisacrylamide was added to chemically cross‐link the copolymers, whereas H‐bonding formed between ‐COOH groups, hydrophobic associations between the alkyl chains and electrostatic interactions between zwitterions all served as physical junctions in the hydrogel networks. Interestingly, these fabricated NSHs could maintain their macroscopic properties and microstructures after several dehydration/hydration cycles.^[^ ^201^ ^]^ Furthermore, disulfide bond is a fascinating covalent bond which can avoid the toxicity of chemical crosslinking agents and response to environmental redox conditions. Base on this, Liu et al. successfully invented smart NSHs dually crosslinked by disulfide bonds and noncovalent interactions, and proved its applicability in anticancer drug delivery and cell encapsulation.^[^ ^202^ ^]^

Kim et al. designed DN NSHs with maximum elastic moduli of 108 MPa and fracture energies of 8850 J m^−2^, by building up IPN between covalently crosslinked poly(2‑hydroxyethyl methacrylate) (pHEMA) and Fe^3+^ crosslinked alginate (Figure 12b–d). Superior to other researches, in vivo non‐swelling capacities of the hydrogels were proved via implantation in rats.^[^ ^203^ ^]^ To optimize the non‐swelling condition, Fan et al. systematically explored the affection of Fe^3+^, gelatin, and AA content on the swelling behaviors of DN gelatin/acrylamide acrylate copolymer‐Fe^3+^ hydrogels. It was found that increasing the Fe^3+^ concentration and AA content could drastically promote the reduction of the SRs of hydrogels.^[^ ^204^ ^]^ These phenomena were attributed to the augmented interactions between Fe^3+^ and ─COOH groups of AA, further confirmed the principle that increasing crosslinking density could suppress swelling.

### 3.2 Realized by Regulating Polymer‐Water Interaction

Introducing stable hydrophobic segments into hydrophilic hydrogel networks is beneficial to drastically decrease the swelling of hydrogel and maintain long‐term stability under liquid condition.^[^ ^205^ ^]^ It can be achieved by copolymerizing hydrophobic monomers with hydrophilic monomers (i.e., amphiphilic block polymer),^[^ ^13^, ^144^ ^]^ or grafting hydrophobic compounds on the hydrophilic hydrogel layers.^[^ ^206^ ^]^ As elucidated above, hydrophobic segments tend to aggregate in water based on hydrophobic association.^[^ ^207^ ^]^ When the hydrophobic aggregates undertake an important role in bridging polymer chains, its major contribution lies in causing a compact network, which is in line with the intention of increasing the crosslinking density (Section 3.1.2). But in some situations, for example, an organic liquid phase immobilized by the hydrogel network, there only occur mild associations in the hydrogel system. So the function of the hydrophobic segments is mainly embodied in offering hydrophobic shielding effects for reducing the interaction between hydrophilic groups and water molecules. Therefore, in this section, we have summarized the methodologies to realize NSHs by making use of the water‐repelling effects of hydrophobic interaction as follows.

#### 3.2.1 Exploiting the Solvent Exchange Approach

When adopting organic solvents to soften the hydrophobic association of hydrogel system, hydrogel with synergistic hydrophobic and hydrophilic network structure may exhibit prominent non‐swelling behavior after immersed in various liquid media, like deionized water, acid and alkali solutions, and even organic solvents. The non‐swelling ability is indispensable for underwater adhesiveness. In addition, the modifiable freezing and boiling point of organic liquids can enable the hydrogel operate across a wide temperature range (especially under sub‐zero temperature).^[^ ^208^ ^]^

**Figure 13:**
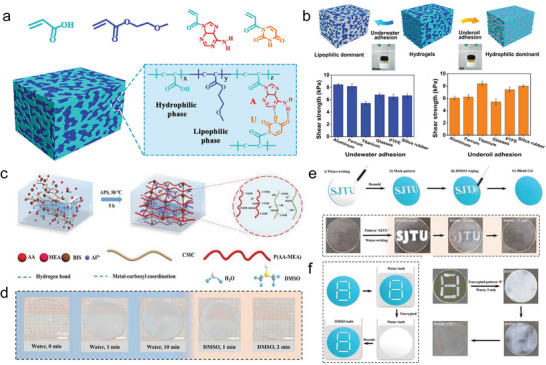
Synthesis and characterization of NSHs based on the solvent exchange approach. a) Schematic structure of the nucleobase pair‐assisted hydrogels. b) Underwater and underoil adhesion mechanism and shear strength of hydrogels on various substrates. Adapted with permission.^[^ ^144^ ^]^ Copyright 2020, Elsevier B.V. c) Illustration of the CMC/P(AA‐MEA)/Al^3+^ DN ionic conductive hydrogel. BIS, *N,N*‐methylenebisacrylamide; APS, ammonium persulfate; d) Photos of the hydrogel from transparency to opacity in water and returning to transparency in DMSO. e,f) Schematic illustrations and photos of information encrypting and decoding. Adapted with permission.^[^ ^13f^ ^]^ Copyright 2021, Wiley‐VCH GmbH.

On this basis, Gao's team have accomplished a class of multifunctional hydrogel with wet‐adhesion performances. First, a non‐swellable underwater adhesive gel was synthesized through copolymerizing AA, butyl acrylate (BA) and acrylated adenine (Aa) in DMSO. After in contact with water, the hydrophobic aggregation of BA was responsible for effectively repelling the water molecules from the gel. Capable of forming physical interaction between substrates and nucleobase molecules Aa, the non‐swelling gel displayed an excellent underwater adhesion for various substrates.^[^ ^209^ ^]^ Later, they replaced single solvent DMSO by DMSO‐water binary solvent, in which the copolymerization of 2‐methoxyethyl acrylate (MEA) and AA occurred, and successfully prepared two kinds of non‐swelling hydrogel sensors. The presence of hydrophilic AA and water molecules in the hydrogels efficiently prevented the corrosion of organic solvents, while the hydrophobic MEA resisted water. And the binary solvent composition also had low freezing point and high stability. All these advantages of bi‐solvents brought the two kinds hydrogels non‐swelling behaviors in not only water but also organic solvents, and wide temperature tolerance from −20 to 70 °C. Differently, one research focused on introducing nucleobase pairs of uracil and adenine to promote the mechanical and adhesive strength of the gels (**Figure** **13**a,b),^[^ ^144^ ^]^ while the other paid more attention to the mechanical robustness as well as outstanding conductive features of graphene.^[^ ^13e^ ^]^ In the end, both kinds hydrogels were proved as wearable sensors for sensing diverse human motions, but the graphene‐based hydrogel conductor could further precisely monitor mechanical deformations in water, chloroform, hexane, and dodecane. Note that the same group also fabricated an anti‐swelling flexible hydrogel via methacrylamide polymerizing in graphene solution. This hydrogel presented brilliant self‐healing capability in various aquatic environments, and outstanding underwater mechanical perception even after healing, which provides inspiration for designing future self‐healing sensors.^[^ ^142b^ ^]^ Similarly exploiting the double‐solvent systems consisting of DMSO and water, Liu et al. designed a DN ionic conductive hydrogel by introducing covalently crosslinked poly(AA‐*co*‐MEA) into CMC skeleton and then both further crosslinked by Al^3+^ (Figure 13c). The hydrogel also demonstrated remarkable mechanical performance, freezing resistance, anti‐swelling and moisturizing retention properties, and high strain‐sensing ability in water and oil environments. But what was noteworthy was that they further appreciated the phenomena that the prepared hydrogels rapidly changed from transparent to opaque in water, due to the microphase separation from solvent exchange (Figure 13d). Utilizing the peculiar optical properties, the hydrogel realized information identification and encryption (Figure 13e,f), which broaden the application range of anti‐swelling hydrogel biosensors.^[^ ^13f^ ^]^

#### 3.2.2 Covering Hydrophobic Layers on Hydrophilic Hydrogel Surfaces

Borrowing ideas from the mammalian skin composed of hydrophilic dermis and hydrophobic epidermis,^[^ ^210^ ^]^ covering hydrophobic layers on hydrogel surfaces was adopted to protect the hydrophilic hydrogels from the invasion of water thus achieve swelling resistance.^[^ ^211^ ^]^ In the meantime, the physical barrier roles of hydrophobic layers can also limit the dehydration of hydrogels, and sustain their flexibility and functionality.^[^ ^212^ ^]^ Therefore, based on the prerequisite that the hydrophobic coatings being robustly bonded to the hydrophilic surfaces without altering the bulk hydrogel structure,^[^ ^213^ ^]^ researchers have generated a variety of meaningful findings.

**Figure 14:**
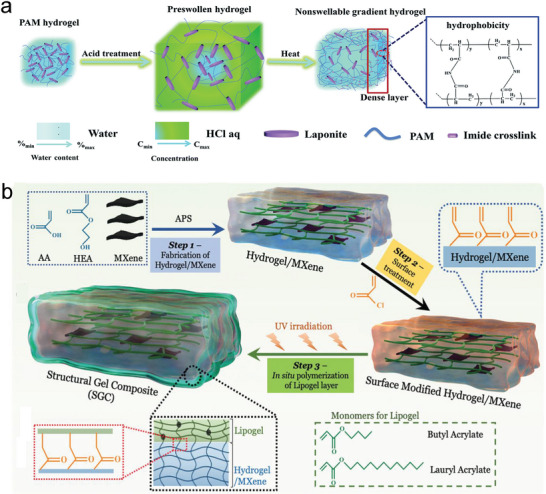
NSHs based on covering hydrophobic layers on hydrophilic hydrogel surfaces. a) Schematic formation of PAM nanocomposite hydrogel with gradient distribution of hydrophobic imide groups. Adapted with permission.^[^ ^214^ ^]^ Copyright 2020, The Royal Society of Chemistry. b) The preparation illustrations of anti‐swelling SGC with robust interface. Adapted under the terms of the Creative Commons CC BY license.^[^ ^210b^ ^]^ Copyright 2022, The Authors.

In one study, a PAM/Laponite nanocomposite hydrogel was designed with gradient distribution of hydrophobic imide groups converted from hydrophilic amide groups via acid‐heat treatment (**Figure** **14**a). The formed gradient structure with a dense cover layer and hydrophobic groups conferred the hydrogel with non‐swelling behavior in water and NaCl solution. And the resultant hydrogel showed high compressive stress of 70 MPa at a higher imidization degree, good fatigue resistance and resilience while at a lower imidization degree.^[^ ^214^ ^]^ Organogels are distinguished from hydrogels by adopting organic liquids as the solvent phase.^[^ ^208^, ^215^ ^]^ In another, hydrogels and organogels were assembled into layered hybrids by copolymerizing organogel monomers with double bonds on hydrogel surface. The outer hydrophobicity/inner hydrophilicity endowed the hybrids excellent swelling resistance and water retention ability.^[^ ^206b^ ^]^ Analogously, a moss‐inspired anti‐swelling organogel–hydrogel skin composed of an adhesive organogel layer and a superoleophobic hydrogel layer, was fabricated via a one‐step wetting‐enabled‐transfer (WET) strategy. Superior to preceding reported superoleophobic materials, the organogel–hydrogel hybrids synchronously possessed robust interfacial adhesion and reliable underwater oil‐repellency, thus could be pasted on various substrates with no need for extra processing. Therefore, the design principle of this organogel–hydrogel skin shed new light on the development of bioinspired interfacial materials with asymmetric properties toward diverse real‐world applications.^[^ ^13g^ ^]^ Interestingly, through encapsulating conductive hydrogel/MXene within a hydrophobic lipid gel (Lipogel), an anti‐swelling and anti‐hydration structural gel composite (SGC) was invented (Figure 14b). The coexisting of strong Lipogel/hydrogel interface and water repellence effect of Lipogel layer empowered the conductive SGC with ultra‐stable mechanical performance in ambient air and water, accurate and dependable underwater sensing ability, and faster responsiveness.^[^ ^210b^ ^]^

### 3.3 Achieved by Incorporating Thermo‐Responsive Polymers

Another emerging strategy for achieving NSHs is based on introducing thermo‐responsive polymers with a lower critical solution temperature (LCST).^[^ ^11^, ^153^ ^]^ Above LCST, the polymers will occur hydrophilic‐to‐hydrophobic transition. The interactions between hydrophobic blocks will cause the hydrogel shrinking, which can counteract the hydrogel's tendency to swell, thus rendering it non‐swellable under physiological conditions (37 °C).^[^ ^140^ ^]^ Among numerous thermo‐responsive polymers, the amphiphilic triblock PEG‐PPG‐PEG (Pluronic) has been widely exploited ascribed to its relatively low LCST.^[^ ^145^, ^216^ ^]^

As described before, Macdougall et al. thought out two routes to prepare non‐swelling PEG click hydrogel and one route was to increase the cross‐linking density. The other route synthesized a 2‐arm thiol‐functionalized thermo‐responsive Pluronic l‐64 and resulted in non‐swelling click hydrogels in PBS at 37 °C, verifying the validity of the method in body temperature.^[^ ^145a^ ^]^ Through covalently cross‐linking di‐acrylated Pluronic F127 (PF127), Shen et al. constructed non‐swelling hydrogel‐based microfluidic chips with unexceptionable mechanical and morphology stability in PBS at 37 °C. The microfluidic chips were autoclavable, which enhanced its practicality. After culturing human umbilical vein endothelial cells in the hydrogel‐based microfluidic chip, a vessel‐on‐a‐chip was established, and the expression of endothelial functions was increased.^[^ ^145b^ ^]^

The concentration or hydrophilic/hydrophobic composition of polymers which exhibit fascinating thermo‐responsiveness, will exert a significant influence on the compaction forces induced by hydrophilic‐to‐hydrophobic transition. When the swelling force of the polymeric network is lower than compaction force, the hydrogel will appear volume shrinkage. The shrinkage effects could find appealing application for hydrogels in biomedicine as well. And the construction and biomedical applications of shrinkable hydrogels will be presented in Section 4.

### 3.4 Biomedical Applications

#### 3.4.1 Tissue Engineering and Regenerative Medicine

##### Wound Healing

HSHs are fairly attractive in hastening full‐thickness skin wound healing. Nevertheless, when HSHs being applied onto human internal soft‐tissue wound sites, the unrestricted swelling in the physiological environment leading to an expanded volume would generate much medical complications. For example, the degradation of mechanical performance and impaired adhesion will decrease the efficacy of the material, expose the wound, and more seriously cause the wound re‐rupture and re‐bleeding.^[^ ^217^ ^]^ In addition, the unexpected swelling can trigger compression damage to surrounding soft tissue, especially in very confined spaces, such as spinal cord,^[^ ^218^ ^]^ oral cavity,^[^ ^219^ ^]^ and cornea defects.^[^ ^220^ ^]^ In contrast, hydrogels with long‐term non‐swelling profile can perfectly avoid these problems,^[^ ^221^ ^]^ thus have gained impressive attention for the healing of wounds.

First of all, NSHs show great prospects for being utilized as wound sealants, because they can retain good interfacial adhesion ability toward various tissues, whether in dry air or water. The leading strategy to acquire attractive bioadhesiveness is employing mussel‐inspired chemistry, which demands abundant catechol groups to form strong multiple H‐bonds with moist biological tissues.^[^ ^222^ ^]^ In view of its abundant catechol groups and great potential to provide numerous crosslinking sites,^[^ ^13^, ^223^ ^]^ polyphenol compound TA has been frequently utilized to yield hydrogel adhesives exhibiting excellent non‐swelling features and wound closure ability. And biocompatible PEG and its derivatives often act as the polymer matrix. For instance, Sun et al. developed an anti‐swelling tissue adhesive via physical interaction between eight‐arm PEG end‐capped with *N*‐hydroxysuccinimide glutarate (PEG‐SG) and TA. The adhesive could tightly attach to porcine tissues, seal rigid vascular artery, and withstand a load of 2 kg. Equipped with syringe‐injectability and self‐healing properties, the adhesive could facilitate self‐rescue and minimal invasive surgery.^[^ ^224^ ^]^ Through introducing TA to form secondary physical cross‐linkage in primary PEGDA covalent network, Du et al. and Chen et al. have both invented a family of multifunctional NSHs. Dissimilarly, the former group also used PF127 diacrylate to self‐assemble to form micelles in water and ‐COOH groups of SA to form intermolecular interactions with tissue surfaces, while the latter group just used PEGDA with substantially higher molecular weight. Although TA is also a natural antibacterial agent,^[^ ^225^ ^]^ only the antibacterial activities of hydrogels fabricated by Du et al. were confirmed.^[^ ^226^ ^]^ In contrast, Chen et al. additionally explored on‐demand detachment, self‐healability, and tailorable topography of their prepared hydrogels.^[^ ^227^ ^]^ In spite of the differences in research priorities, in full‐thickness skin wound models, the wound closure ability of the above three TA‐based hydrogel adhesives were all better than that of sutures and commercial tissue adhesives. Therefore, these non‐swelling hydrogel adhesives hold great promise for wound healing. And the discrepant design principles can inspire researchers to employ other different polymeric materials to expand the family of TA‐containing hydrogel adhesives. Interestingly, new critical insights into designing adhesive hydrogels have been provided by Yu's team. They have prepared novel non‐catechol‐based injectable adhesive NSHs via the dynamic boronic ester bonds formed between phenylboronic acid groups from poly(*N,N*‐dimethylethylenediamine‐*g*‐3‐bromomethylphenylboronic acid) phosphazene (PPBA) and hydroxyl groups from PVA. Electrostatic interactions conferred the hydrogels excellent antibacterial property, while π‐π/cation‐π interaction and H‐bonding enabled them stably adhere to tissue surfaces in water. Moreover, in vivo assays manifested that the hydrogels could efficiently promote hemostasis and accelerate wound healing. Thus, the hydrogel adhesives with multi‐functions possess significant application value in treating acute trauma or surgical injuries.^[^ ^228^ ^]^

**Figure 15:**
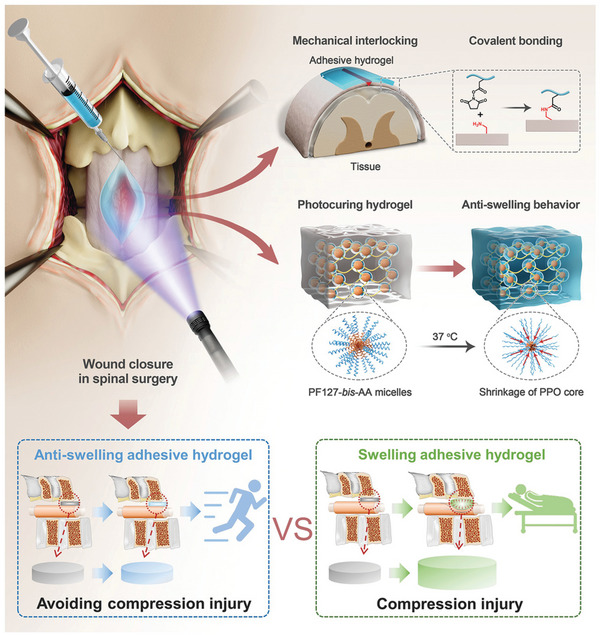
NSHs utilized for wound sealing in spinal surgery. Schematic diagram on the anti‐swelling hydrogel with injectability and rapid‐adhesion property. Adapted with permission.^[^ ^13h^ ^]^ Copyright 2022, Wiley‐VCH GmbH.

**Figure 16:**
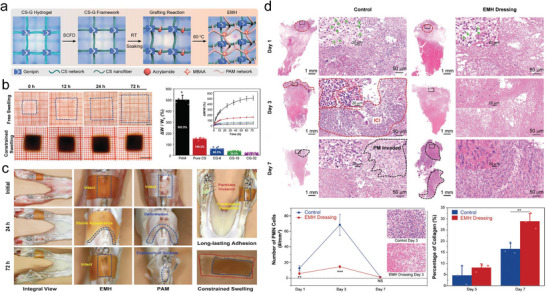
NSHs utilized as intraoral wound dressing. a) Preparation of the genipin‐crosslinked chitosan (G‐CS) meshwork and ECM‐mimicking hydrogel (EMH). SCFD, supercritical fluid drying; RT, room temperature; MBAA, *N,N*′‐methylene bisacrylamide; b) volume and weight change of the EMH and PAM hydrogel in PBS. c) Comparison of PAM and EMH hydrogel dressings in protecting oral extraction defects. d) Histological analysis of inflammatory cell infiltration (ICI) and provisional matrix (PM) at tooth extraction socket of the control and EMH‐treated groups. Adapted with permission.^[^ ^219^ ^]^ Copyright 2022, Wiley‐VCH GmbH.

In addition, several advanced findings have constructed NSHs and verified its superiority in treating some internal wound defects. For instance, based on thermo‐sensitive shrinkable PF127 nano‐micelle cross‐links, Zhao et al. fabricated an anti‐swelling hydrogel with both injectability and rapid‐adhesion property for dura sealing without oppressing spinal cord (**Figure** **15**). The hydrogel could undergo ultrafast gelation within 2 s of UV illumination and achieve wet adhesion via small molecular adhesive moieties acrylic acid *N*‐hydroxysuccinimide ester.^[^ ^13h^ ^]^ Through imitating the structure of peritoneum, a biocompatible Janus porous PVA (JPVA) hydrogel with anti‐swelling property was developed to repair internal abdominal wall defects. The JPVA hydrogel exhibited densely porous bottom‐surface and loose ECM‐like porous top‐surface, which improved fibroblast tissue growth and minimized visceral adhesion. And the long‐lasting mechanical strength of the anti‐swelling hydrogel resisted the maximum abdominal pressure in the humid internal environment. Thus, the JPVA hydrogel indicated superior abdominal wall defect treatment than commercially available polypropylene and Parietex composite meshes.^[^ ^229^ ^]^ Borrowing ideas from ECM, Wu et al. invented a family of hydrogel dressings for managing tooth extraction sockets (Figure 16). The antibiotics‐loaded hydrogel consisted of a CS fibrous framework and a high ductile polymerized long‐chain PAM hydrogel (**Figure** **16**a). Resulting from the crosslinking by genipin and deprotonation by alkali solution, the rigid CS fibrous meshwork was responsible for providing mechanical support and preventing the long‐chain PAM network from swelling (Figure 16b,c). Importantly, the hydrogel dressing exhibited long‐acting protection for intraoral wounds and enhanced wound healing on a canine tooth extraction model (Figure 16d), thus were promising candidates as intraoral wound dressings.^[^ ^219^ ^]^

In general, recent NSHs create a paradigm shift in developing adhesives for wound sealing due to their persistent wet‐adhesion performance. But beyond that their potential as dressing materials in some specific internal wound sites have gradually attracted great interest as well. This trend will considerably enlarge the biomedical application range of NSHs, but accordingly put forward great requirements in its physical performance and biological activities.

##### Other Tissue Regeneration

NSHs with assembly of outstanding physical and biological properties, which can be preserved for a long time, have gained impressive attention for bone tissue engineering. For instance, non‐swelling methacrylated HA hydrogel scaffolds crosslinked by PF127 diacrylate micelle, showed the capability of promoting thyroid cartilage defect regeneration.^[^ ^230^ ^]^ Another excellent non‐swelling hydrogel integrating mechanical stability, conductivity, and photothermal antibacterial activities was conducted based on double‐bond radical polymerization and host‐guest complexation between GelMA, acryloyl‐β‐CD, and β‐CD‐functionalized rGO. The biocompatible hydrogels could afford the regeneration of in vivo skull defect.^[^ ^231^ ^]^ Meaningfully, a super‐strong bioinspired meniscus substitute was produced by Zhang et al. The scaffold was constituted by a 3D printed radially and circumferentially oriented poly(e‐caprolactone) (PCL) fiber matrix that replicated collagen fibers in meniscus, and H‐bonding reinforced non‐swelling poly(*N*‐acryloyl glycinamide) (PNAGA) hydrogel that shouldered the obligation of withstanding axial compressive loads as proteoglycan. Benefiting from the biomimetic microarchitecture, the PCL‐PNAGA scaffolds achieved ultra‐high Young's moduli of 20.15 MPa in circumferential direction and 10.43 MPa in radial direction, compressive modulus of 1.11 MPa and tearing energy of 17.00 kJ m^−2^. Therefore, the PCL‐PNAGA meniscus scaffold efficiently protected in vivo cartilage against wearing, and inhibited the progressive of osteoarthritis.^[^ ^232^ ^]^ Besides, NSHs have been gradually served as space‐filling grafts in tension‐freely internal tissues, like artificial vitreous body,^[^ ^143^, ^233^ ^]^ and craniofacial soft tissues. Injectable non‐swelling hydrogel holds potential in minimally invasive tissue engineering.^[^ ^234^ ^]^ Equipped with ideal antibacterial and reactive oxygen species scavenging capacities induced by Ti_3_C_2_ MXene additives, anti‐swelling hydrogel could also promote diabetic tissue engineering.^[^ ^235^ ^]^ These advances will be great inspirations for extending the application range of NSHs in tissue regeneration.

#### 3.4.2 Bioelectronics

Hydrogels with a suite of intriguing performances such as tissue‐like mechanical properties, outstanding stretchability, and conductivity, are of great significance in soft bioelectronics industry.^[^ ^236^ ^]^ However, the aggressive swelling of hydrogels will reduce their mechanical robustness and electrical conductivity, which largely hindered their practical applications in complex underwater conditions.^[^ ^237^ ^]^ As a consequence, there is a great promise for applying NSHs as bioelectronic devices.^[^ ^238^ ^]^ At present, the functional element (i.e., conductivity) of NSHs can be realized by utilizing either conductive organic materials like zwitterionic polymers or ionic liquid,^[^ ^239^ ^]^ or inorganic materials like metal ions.^[^ ^240^ ^]^

As elucidated before, the mechanism of DN strategy have been widely employed to synthesize NSHs. Benefiting from the ease of simultaneously assembling high strength, stretchability, and high conductivity, DN NSHs provide an important direction for the modernization of strain sensing. Naturally, several researches in related fields have set their sights on this strategy. For instance, the work of Ren et al. presented a DN non‐swelling hydrogel constituted by PVA and P(SBMA‐*co*‐HEMA). Importantly, the incorporation of zwitterionic SBMA brought a high conductivity (up to 4.58 S m^−1^). Together with excellent mechanical and anti‐swelling properties, the hydrogel soaked in water for 24 h showed high sensitivity, whose gauge factor (GF) were 1.434, 2.448, and 3.356, respectively in strain range of 0–100%, 100–200%, and 200–300%. Besides, when subjected to loading and unloading, the hydrogel sensor displayed short response time of 130 ms and recovery time of 200 ms, and did not suffer any attenuation upon cycling for 1000 times at 50% strain.^[^ ^239a^ ^]^ Another non‐swelling DN hydrogel with stable mechanical properties was developed by adding CS into P(AA‐MEA)‐Fe^3+^ network. After immersed in water for a week, the resulting hydrogel demonstrated high strain sensitivity (GF 1.60) at first 100% strain and accuracy (1‐5% strain), as well as long‐term stability (100 cycles at 50% strain).^[^ ^240b^ ^]^ Intriguingly, Zhang et al. fabricated a biocompatible cellulose biomimetic hydrogel (CBH) with enhanced toughness, stretchability, modulus, self‐stiffness, and elasticity. The CBH composed of a non‐swelling fiber framework derived from the crystallization behavior of cellulose and a chemically crosslinked PAM, which respectively represented the collagen fibers and elastic elastin of skin structures. What's more, through in situ polymerization of aniline within CBH, a conductive CBH hydrogel biosensor was performed with GF 1.7 in the strain range of 90–600%.^[^ ^47^ ^]^ In sum, these hydrogel biosensors can realize the real‐time monitoring of human motions under a wet environment, which proved the validity of adopting DN strategy to generate high‐performance non‐swelling hydrogel biosensors.

In order to meet the practical applications of hydrogel bioelectronics, tremendous efforts have been devoted to endowing conductive NSHs with additionally fascinating functionalities in terms of self‐healing and shape memory,^[^ ^241^ ^]^ temperature‐tolerant,^[^ ^241^, ^242^ ^]^ adhesiveness,^[^ ^243^ ^]^ and anti‐bacterial activity.^[^ ^244^ ^]^ These desirable characteristics will improve the reliability and service life of hydrogel bioelectronic devices, as well as expand their applications in harsh environments.

Relying on crystal microphase crosslinking formed by acrylonitrile segments, He's team fabricated sustainable and easy‐processing acrylonitrile copolymer hydrogels with high tensile stress (23 MPa) and fracture energy (592 J m^−2^). With prominent anti‐swelling properties, the physically crosslinked hydrogel could remain stable in water for more than 60 days. And it was found that the hydrogel also has obvious pH‐induced shape‐memory. After immersed in ZnCl_2_ solution, the hydrogel was further strengthened and toughened, and significantly, its electrical conductivity was greatly improved (9.11 S m^−1^), which enabled it as strain sensor with high sensitivity, good linear response, and reliable stability.^[^ ^241a^ ^]^ Through concurrently adding zwitterionic proline, Al^3+^, and conductive poly(3,4‐ethylenedioxythiophene):polystyrene sulfonate (PEDOT:PSS) into vinyl hybrid SiO_2_ NPs cross‐linked PAA networks, Feng et al. obtained conductive hydrogels with extraordinary stretchable (1769%), self‐healing, anti‐dehydration, and anti‐swelling abilities. The hydrogel‐based strain sensor displayed high strain sensitivity (GF 3.07), wide sensing range (5–700% strain), stability (200% strain for 800 cycles), and precise human movements, even in water/organic solvents and at −40 °C. More interestingly, the hydrogels could achieve anti‐freezing shape memory and cyclic information recording/erasing under external stimuli.^[^ ^241b^ ^]^ In turn, Wu et al. reported a salt‐percolated method to synthesize NSHs with distinguished anti‐freezing and drying abilities by incorporating LiBr. It was discovered that 50 wt% LiBr‐percolated PAM/carrageenan hydrogels could retain superior stretchability and conductivity (12 S m^−1^) even in freezing condition of −78.5 °C and open air for one year. Significantly, the hydrogel concurrently showed high sensitivity to temperature and strain within wide ranges, qualifying it as high‐performance multifunctional sensors. And it could monitor various physiological signals as well.^[^ ^242^ ^]^

**Figure 17:**
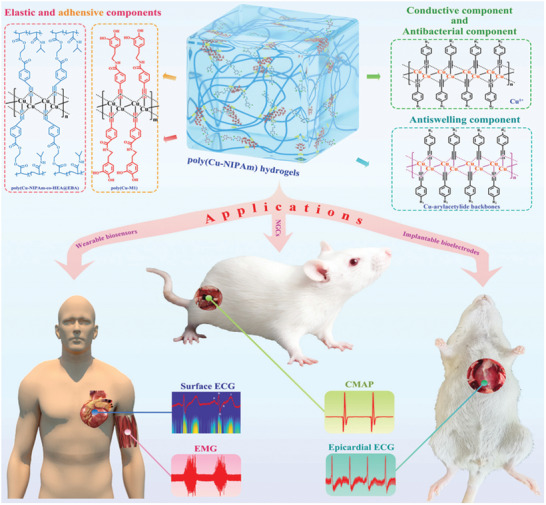
Conductive NSHs utilized as implantable sensors for bioelectronics. Demonstration of the preparation scheme and bioelectric monitoring applications of poly(Cu‐NIPAm) hydrogels. CMAP, compound motor action potential. Adapted with permission.^[^ ^244b^ ^]^ Copyright 2022, Wiley‐VCH GmbH.

Conducting NSHs are also promising candidates in the field of bioelectric monitoring. Compared to strain sensors, the bioelectrical electrodes demand higher accuracy and stability during detection process.^[^ ^239b^ ^]^ And an ideal bioelectrical conductor also requires excellent biocompatibility and reliable underwater adhesion. Under this premise, Guo et al. achieved a non‐swelling hydrogel electrode with high conductivity (10 S cm^−1^) and stretchability (150%), through concentrating a high content (5.5 wt%) of PEDOT:PSS within a poorly crosslinked PVA network. Especially noteworthy was that they further achieved robust and rapid bioadhesiveness via *N*‐hydroxysuccinimide coupling reaction with tissue surfaces.^[^ ^243b^ ^]^ However, it has not been confirmed yet that whether implementing adhesion through covalent crosslinking will induce chemical contamination and organ damage or not. Therefore, Han et al. further manufactured a class of conductive NSHs, which exhibited good adhesion performance via forming noncovalent interactions with diverse substrates, such as hydrogen bonds and electrostatic interactions. Cation‐π interactions between graphite and zwitterionic polymers were the driving forces for forming the hydrogels. Both kinds electrical bioadhesive NSHs demonstrated desired in vitro and in vivo biocompatibility, tissue‐like mechanical and electrochemical performances. And most importantly, they can spontaneously adhere to muscles for high‐quality electrophysiological signal recording, and a sciatic nerve for reliable electrical stimulation.^[^ ^243a^ ^]^ Moreover, Sun et al. reported a bioelectrical gel electrode by introducing choline acrylic‐based bioionic liquid and TA into the polymerization of AA and BA in DMSO. With the assist of swelling resistance, the physical interactions between the gel and substrate surface endowed the gel electrode strong underwater adhesion, while bioionic liquid gave long‐lasting high conductivity and high‐sensitivity bioelectric monitoring even in wet environment. The gel electrode exhibited very low impedance (<10 Ω) and nearly inappreciable noise in static and dynamic monitoring, thus can timely obtain high‐quality electrocardiogram (ECG) and electromyographic (EMG) signals in calm or exercise underwater conditions.^[^ ^239b^ ^]^ Especially, Xia et al. additionally imparted antibacterial effect into hydrogel electrodes through introducing poly(Cu‐arylacetylide) component, which also allowed good electron conductivity, high swelling resistance, adhesion and biocompatibility (**Figure** **17**). The hydrogels were able to reliably record surface and epicardial ECG, EMG, as well as transmit neural signals. Taken together, this electroactive hydrogels assembling desirable functionalities not only hold profound potential for use as implantable electrodes and wearable sensors, but also provided valuable concepts for the design of bioelectronics by replacing Cu (I) in polymer chains by other metal ions.^[^ ^244b^ ^]^ All these multifunctional hydrogel successfully overcome the shortcomings of swelling of current commercial electrodes, providing new paths for underwater bioelectric monitoring. Besides, it will be possible for them to advance the future development of implantable bioelectronics and clinical electronic therapeutics.

## 4 Shrinkable Hydrogels

Shrinkable hydrogels (SHs) are a kind of hydrogels which will deswell when subjected to external fields, like temperature, pH, or ion conditions. The deswelling process causes a reduction in macroscopic volume of hydrogel, that is, shrinking. This responsive‐shrinking behavior highly depends on polymer composition of the hydrogel. Thus, with the aim to understand the shrinking mechanism and fabrication methods of SHs convenient for further study and application, it is meaningful to summarize and analyze the latest developments of SHs (**Table** **4**).

**Table 4:** Brief summary of the constructions, properties, and biomedical applications of SHs

Constructions	Hydrogel systems	Maximum shrinkage ratios	Properties	Biomedical applications	Ref.
Based on thermo‐responsive polymer	PNIPAm, alginate, Ag NPs	80% after 3 h at 37 °C	Bioadhesive, tough, highly stretchable, thermo‐responsive shrinkage, antimicrobial	Wound closure and healing	[259]
	P(NIPAm‐*co*‐THMA), GelMA, ε‐PL/GA, lidocaine, Ag NPs	43.3% after 1 h at 37 °C	Double‐sided, thermo‐responsive shrinkage, great flexibility, self‐healing, underwater bioadhesion, antimicrobial	Joint wound closure and healing	[14b]
	PNIPAm‐AA, QCS‐CD, PPY	53% after 2 h at 37 °C	Self‐healing, bioadhesive, elastic, thermo‐contraction, antioxidant, hemostatic, antibacterial	Wound closure and MRSA‐infected wound healing	[261b]
	PNIPAm, HA	70% after 6 h at 37 °C	Thermo‐contraction, bio adhesive	Wound closure and healing	[281]
	PNIPAm, GelMA	53.1% after 1 h at 37 °C	Thermo‐responsive shrinkage, biocompatible	Scaffolds with engineered microscale vasculatures	[264]
	PEI, 4‐armed P(DMAEMA‐*co*‐HEMA‐*t*‐BAA), PDA NPs, DOX	58% under NIR irradiation (808 nm, 1 W cm^−2^, 5 min)	Injectable, self‐healing, photothermal, thermo‐responsive shrinkage	Anti‐cancer drug delivery and cancer thermotherapy	[270b]
	P(NIPAm‐+‐BBVIm), CS, carbon nanotubes, ketoprofen	30% after switching on/off electrical and thermal signal within 5 min	Electrical and photothermal responsive, thermo‐ responsive shrinkage, biocompatible	Switchable drug delivery	[271]
	Pluronic F127‐ end functionalized α‐lipoic acid, BSA	Not involved	Reduction responsive, thermo‐responsive shrinkage, self‐healing, sustained drug release, biocompatible	Drug delivery	[267]
Based on other responsive polymers	Bombyx mori silk fibroin, CMC, gelatin	50% after 24 days	Injectable, resilient, self‐stiffing, and contractile	Cartilage tissue engineering	[14a]
	Catechol‐modified CS, dibenzaldehyde‐terminated PEG_2000_	50% after 24 h in PBS	Self‐healing, wet adhesion, saline‐induced contractile, biocompatible, hemostatic	Wound closure and healing	[14d]
	CS, HAMA	90.3% at pH 4.7	3D printable, charge compensation‐induced shrinkage, cytocompatible	Higher‐resolution scaffolds	[14e]
	Carboxymethyl chitin, Fe_3_O_4_, 5‐fluorouracil	29.9% after 3 h at pH 1.0	pH and magnetic sensitive, pH‐induced shrinkage	pH‐controlled drug release	[273]

### 4.1 Based on Thermo‐Responsive Polymers

At present, the most widely investigated SHs are thermo‐responsive hydrogels. And PNIPAm is the most commonly employed thermo‐responsive polymer for fabricating SHs, due to their sharp phase transition at LCST (about 32 °C), and facile preparation.^[^ ^245^ ^]^ As temperature exceeds the LCST, the transition from hydrophilicity to hydrophobicity can repel water and induce volume shrinkage of PNIPAm‐based hydrogels.^[^ ^246^ ^]^ The temperature is close to human body temperature and can be adjusted in accordance with therapeutic requirements,^[^ ^247^ ^]^ thereby is very well suited for biomedical applications. Therefore, there have been numerous examples of PNIPAm‐based SHs over the last five years.

Through introducing biocompatible poly(l‐lactic acid) (PLLA) and poly(d‐lactic acid) (PDLA) into PNIPAm, the PNIPAm‐*g*‐PLLA and PNIPAm‐*g*‐PDLA copolymer hydrogels were obtained via the hydrophobic PLLA/PDLA domains serving as physical crosslinking sites. The stereocomplex crystallization between PLLA and PDLA bestowed the hydrogels with higher mechanical robustness and better solvent resistance than enantiopure examples. With the crystalline structure being easily controlled by changing the ratio of PLLA/PDLA enantiomeric blocks, the volume shrinkage at high temperatures could be controlled in turn.^[^ ^248^ ^]^ Interestingly, in another contribution, a synthesized core‐crosslinked star‐shaped polymer with multiple PNIPAm arms, were exploited as crosslinker for the formation of PAM gel. The star‐crosslinked gel showed unique thermo‐responsive swelling behavior and mechanical toughening due to the effective function of the PNIPAm arms.^[^ ^249^ ^]^

PNIPAm‐based hydrogels with additional functionalities can be achieved through adding functional materials further, which may promote the application of the hydrogels. In order to acquire both reversible volume shrinkage behavior and redox‐responsiveness, Park et al. blended PNIPAm hydrogel with R825‐loaded carbon dots. Notably, the hydrogels could generate a higher photothermal temperature after GSH treatment and then effectively kill cancer cells in vitro. Thus, they can be applicated for cancer therapies.^[^ ^250^ ^]^ Equally interesting was the 4D printed‐smart breathing hydrogel actuators reported by Zhao et al., which was constituted by a spinach leaf‐extracted nanothylakoid membrane for photothermal conversion and catalytical O_2_ evolution, a PNIPAm network for generating deformation forces by swelling/shrinkage (rehydration/dehydration), and an asymmetric PNIPAm/PAM bilayer structure to magnify the mechanical motions. The actuator can dynamically bend/unbend, and generate O_2_ upon thermal stimulus or laser emission, which opened novel avenues for the future progress of intelligent hydrogel systems.^[^ ^251^ ^]^ Moreover, several research groups have simultaneously realized sensing and actuation of PNIPAm hydrogels with photothermal responsiveness, via the introduction of conductive materials such as polyaniline,^[^ ^252^ ^]^ and silver flakes.^[^ ^253^ ^]^ Additionally, magnetic nanoparticles may be added to the PNIPAm hydrogel matrices, in order to realize shape morphing of hydrogels via giant contraction upon external stimuli of alternating magnetic field.^[^ ^254^ ^]^

### 4.2 Based on Other Responsive Polymers

Also, the inclusion of other responsive polymers into a hydrogel can lead to mediated deswelling of hydrogels. SF can spontaneously transform from random coil to β‐sheets under physiological conditions, which induces shrinkage of SF‐based hydrogels.^[^ ^255^ ^]^ According to this, a triple network injectable hydrogel of Bombyx mori SF, CMC, and gelatin, demonstrated reinforced stiffness and contraction after incubated in PBS.^[^ ^14a^ ^]^ In addition, the counterions could shield the electrostatic repulsion between protonated CS chains, thereby inducing physical chain entanglement and the formation of physical junctions via hydrogen bonding.^[^ ^256^ ^]^ With this rationale, a self‐healing CS hydrogel with volume shrinkage behavior in simulated internal conditions of human body, was prepared via Schiff base reaction between catechol‐modified CS and dibenzaldehyde‐terminated PEG_2000_.^[^ ^14d^ ^]^ In a similar way, other polyelectrolytes, whether natural or synthetic, all have the possibilities to construct SHs via the reduction of electrostatic repulsion and osmotic pressure,^[^ ^113^, ^257^ ^]^ which are worth studying in the near future.

### 4.3 Biomedical Applications

#### 4.3.1 Tissue Engineering and Regenerative Medicine

##### Wound Healing

Traditional hydrogels passively aid wound healing by offering a moist wound microenvironment and preventing bacterial infection. However, they are incapable of actively driving wound closure. Borrowing ideas from the healing process of embryonic wounds, which involve the formation of actin cables in the cells at the edge of wounds that actively contract and exert force to pull the wound edge together,^[^ ^258^ ^]^ SH dressings open a new era in wound care. The mechanoactive biological approach of SH dressings can synchronously drive wound closure and enhance wound healing, which creates a two‐pronged tactics for wound treatment.

To quickly transmit the stress to dynamic wound edges, strong tissue adhesion is required. To this end, recent developments have mainly focused on developing PNIPAm‐based thermo‐contracting adhesive hydrogel wound dressings.^[^ ^14c^ ^]^ These dressings can adhere strongly to the skin and exert contractile forces to assist wound closure, triggered by the intrinsic temperature change after placed onto the skin. And they have multiple bio‐functions for supporting wound healing as well.

**Figure 18:**
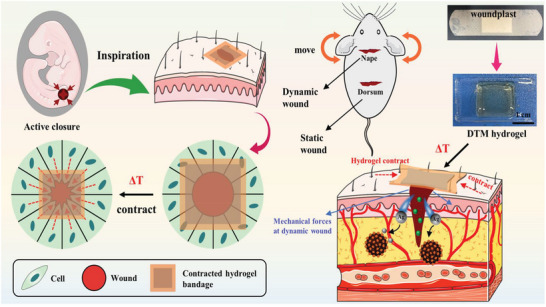
SHs simultaneously accelerate wound closure and wound healing. Diagram of mechanoactive hydrogel dressings enlightened by the healing process of embryonic wounds, and the DTM hydrogel speeding up the contraction and healing of dynamic and static wound. Adapted with permission.^[^ ^14b^ ^]^ Copyright 2022, American Chemical Society.

**Figure 19:**
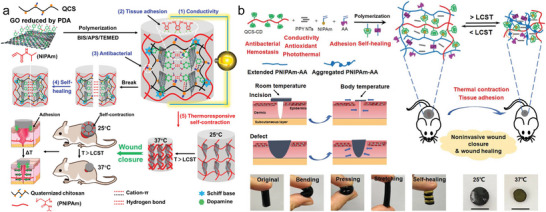
Two kinds of multifunctional PNIPAm hybrid hydrogel dressings enhanced wound healing through thermo‐responsive self‐contraction. a) QCS/rGO‐PDA/PNIPAm hydrogel. Adapted with permission.^[^ ^261a^ ^]^ Copyright 2020, American Chemical Society. b) PNIPAm‐AA/QCS‐CD/PPY hydrogel. Scale bar: 1 cm. PPY NTs: polypyrrole nanotubes. Adapted with permission.^[^ ^261b^ ^]^ Copyright 2022, Wiley‐VCH GmbH.

The pioneering work comes from Blacklow et al., which created a significative paradigm for mechanized wound treatment. In the study, a hybrid PNIPAm‐alginate hydrogel framework was formulated for high stretchability, toughness, and tissue adhesion. And Ag NPs were added into the network to provide antimicrobial function. It was validated that the adhesive dressings were efficient in contracting skin wounds and accelerating wound healing.^[^ ^259^ ^]^ Moreover, through mimicking the structure of available woundplast, Yang et al. invented a double‐sided thermo‐responsive mechanoactive (DTM) hydrogel which combined great flexibility, self‐healing, underwater bioadhesion, and antimicrobial activities (**Figure** **18**). Strikingly, this hydrogel bandage could not only enhance the repair of static wound defects but also dynamic ones. In conclusion, this method demonstrated strong potential in joint wound management and tissue remodeling.^[^ ^14b^ ^]^ Exploiting GO to load Ag NPs thus reduce its aggregation, Fan et al. acquired hydrogel that could effectively inhibit against *E. coli* and *S. aureus*. This attribute, plus with the high contractility of the hydrogel under NIR irradiation deriving from photothermal effect of GO, made the wound healing rate of the composite hydrogel reach 100% within 15 days, far higher than pure PNIPAm hydrogel.^[^ ^260^ ^]^ Differently, Guo et al. introduced QCS to enhance the antibacterial capacity of PNIPAm hydrogel. As shown in **Figure** **19**, they designed two different kinds of multifunctional PNIPAm hybrid hydrogel dressings: QCS/rGO‐PDA/PNIPAm hydrogel via Schiff base, hydrogen bonds, and cation‐π interaction, PNIPAm‐AA/QCS‐CD/PPY hydrogel via host‐guest interaction and hydrogen bonds.^[^ ^261^ ^]^ Both kind hydrogels had excellent thermo‐responsive shrinkage and tissue adhesion abilities, as well as multiple biochemical functions that facilitated wound healing, including self‐healing, antioxidant and intrinsic/photothermal antimicrobial activity. Hence, the hydrogel dressings simultaneously promoted wound closure and healing with higher granulation tissue thickness, collagen disposition, and advanced angiogenesis. What's more, the PNIPAm‐AA/QCS‐CD/PPY hydrogel also showed enhanced skin regeneration when treating methicillin‐resistant *S. aureus*‐infected wounds with photothermal treatment, which have great practical value for anti‐infection.^[^ ^261b^ ^]^

In a nutshell, there is a great possibility for the mechanotherapeutic methods subverting the traditional treatment approaches on wound healing. But before that, further investigations are needed to carry out. For example, the contraction forces need to be optimized to prevent excessive wound tension. In addition, in view of varied temperature at different sites on the body,^[^ ^262^ ^]^ and at different ambient temperatures, it is exigent to develop hydrogels with the capacity to contract over a wide temperature range or independent of temperature, for example, saline‐induced contractile CS hydrogel.^[^ ^14d^ ^]^

##### Other Tissue Regeneration

**Figure 20:**
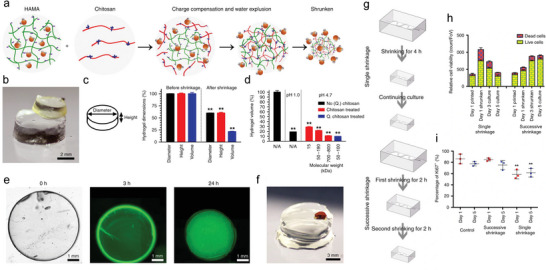
Exploiting the shrinkage behavior of SHs to acquire higher‐resolution hydrogel‐based scaffolds for tissue engineering. a–f) The shrinking behavior of the hydrogel. HAMA, hyaluronic acid methacrylate. g–i) Biocompatibility of the single and successive shrinkage in the existence of living cells. Adapted with permission.^[^ ^14e^ ^]^ Copyright 2020, The Authors.

The shrinkage behavior of hydrogel has made great contributions in precise fabrication of higher‐resolution hydrogel‐based scaffolds for tissue regeneration. For instance, utilizing the shrinkage and dehydration of hydrogel scaffolds, Oran et al. achieved controllable nanoscale feature sizes.^[^ ^263^ ^]^ More recently, Gong et al. not only enhanced resolution of 3D printed hydrogel purely by charge compensation‐induced shrinking (**Figure** **20**a–f), but also indicated that successive shrinking could keep living cells viable in the printed hydrogel in comparison with a single, longer shrinking step (Figure 20g–i).^[^ ^14e^ ^]^ The abovementioned significant progresses in fabricating hydrogel scaffolds open new opportunities for the creation of microscale vasculatures (MSVs) within the engineered tissues. As a result, a facile strategy for MSV production was presented by Li et al., which relied on sacrificial alginate fibers and thermo‐responsive shrinkage of hydrogel scaffolds composed of PNIPAm and biocompatible GelMA. The biocompatibility of the scaffold was proved by a formative HUVEC monolayer and high viability of the embedded osteosarcoma cells. Additionally in vivo experiment validated the efficacy of MSVs in providing a site for the perfusion of host vessels. Hence, the MSVs have strong potential for narrowing the gap between vascular construct and tissue function.^[^ ^264^ ^]^ Furthermore, hydrogel contraction may generate surface microstructures which are conducive to cell attachment,^[^ ^265^ ^]^ as well as promote cellular condensation to enhance chondrogenic differentiation.^[^ ^14a^ ^]^

#### 4.3.2 Drug Delivery

Different from the porous HSHs releasing drugs from molecular diffusion processes, SHs are highly appreciated in drug release by the combined effect of free diffusion and extrusion. This is due to that volume shrinkage is conducive to squeeze out the entrapped biomolecules, thus triggering fast release rate.^[^ ^266^ ^]^ But there is also an exceptional circumstance. When delivering biomacromolecules like BSA, the shrinkage behavior of the hydrogel will increase the difficulty of drug movement. This phenomenon can greatly inhibit the burst release of drug and promote sustained release, thus enhancing the bioavailability of drug molecules at target site.^[^ ^267^ ^]^ Hence, in either cases, the shrinkage behavior have gathered much attention in engineering the release behavior of hydrogel drug carriers. And the fabricated hydrogels are usually dual as well as multi‐stimuli responsive. Stimuli to which the systems react could be intrinsic cues such as pH,^[^ ^268^ ^]^ temperature,^[^ ^268a^ ^]^ and redox,^[^ ^269^ ^]^ or extrinsic cues such as and light,^[^ ^270^ ^]^ electric field,^[^ ^271^ ^]^ and magnetic field,^[^ ^272^ ^]^ or the combination of both.^[^ ^273^ ^]^

SHs in response to intrinsic stimulus are generally considered as intelligent platforms for localized delivery of chemotherapeutic agents. In this regard, a pH/heat dual‐sensitive hydrogel was developed via a synergy of PAA/CS‐Nb polyelectrolyte network and CS‐Nb/bisTz‐PNIPAm chemical crosslinking network. The porosity resulting from the IEDDA click reaction, pH‐responsiveness from PAA and thermo‐responsive shrinkage from PNIPAm, bestowed the hydrogels with high drug load content, colon selectivity and ameliorative drug release capacity. Moreover, the biodegradability and biocompatibility of the hydrogels fostering their use as carriers in colon‐targeted drug delivery.^[^ ^274^ ^]^ In another contribution, the inclusion of succinylated cellulose nanocrystals at different DS ratios and PNIPAm achieved IPN hydrogels with pH and thermo‐responsive characters, which could be used to deliver Famotidine, an active material for reliving the stomach pain.^[^ ^275^ ^]^

**Figure 21:**
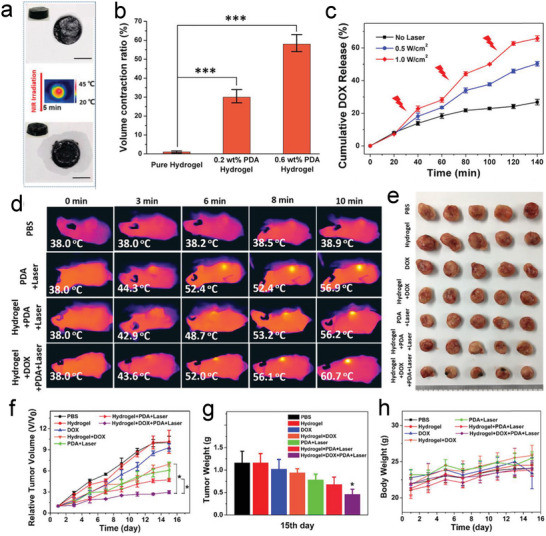
SHs simultaneously perform hyperthermia and deliver DOX for treating cancer. a) Macroscopic views and temperature distribution of the hydrogel samples before and after NIR irradiation. Scale bar: 1 cm. b) Volume contraction ratio of hydrogels with different PDA NPs contents. c) Cumulative DOX release curves of the hydrogels with/without NIR irradiation. d) Temperatures of mice tumors under different treatments within 10 min. f) Relative tumor volume and h) body weights of mice under different treatments within 15 days. e) Photographs and g) weights of mice tumors under different treatments at the 15th day. Adapted with permission.^[^ ^270b^ ^]^ Copyright 2020, American Chemical Society.

Exogenous cues can be used to remotely control the contraction of hydrogel, which is beneficial for the intermittent on‐demand drug release.^[^ ^270a^ ^]^ Therefore, several researchers have prepared photoactive hydrogel DDS through incorporating photothermal agents. These DDS have high competitiveness in treating cancer because of their ability to perform hyperthermia and chemotherapy in concurrently. Concretely, Yuan's team prepared injectable self‐healing hydrogel with volume shrinkage behavior under NIR laser irradiation (**Figure** **21**a,b). The hydrogel loaded with DOX could be noninvasively administered at the 4T1 tumor. And after NIR irradiation, the tumor cells could be effectively killed by the synergistic effect of local hyperthermia induced by photothermal conversion of PDA NPs, and DOX squeezed out from hydrogel shrinkage (Figure 21c–h).^[^ ^270b^ ^]^ Compared to PDA NPs, carbon nanotubes (CNTs) not only have excellent photothermal conversion performance,^[^ ^276^ ^]^ but also electric conductivity,^[^ ^277^ ^]^ which could be combined to further finetune the drug release. For this basis, injectable photoelectron‐active DDS were developed based on silk and folic acid (FA)‐decorated single‐walled CNT (SWCNT). In vivo studies revealed that applying NIR laser and/or a small electrical field could cause the DOX release from the SWCNT‐FA reservoir and the apoptosis in the cancer cells. At the same time, there appeared no cardiotoxicity after the localized DOX delivery.^[^ ^270a^ ^]^ In addition, with the assembly of CNTs as the core and electrical/thermal conductor, CS as the shell and hydrophilic dispersant, and poly(NIPAm‐*co*‐BBVIm) as the drug carrier and a temperature‐responsive copolymer, another electrical, and photothermal‐responsive 3D hydrogels were achieved. The 3D hydrogel could deliver about 37% of ketoprofen for treating musculoskeletal pain, during ≈30% shrinkage after switching on/off electrical and thermal signals.^[^ ^271^ ^]^ Furthermore, as a fellow electrical/thermal conductor, MXene has broad utility in constructing multi‐responsive DDS as well.^[^ ^278^ ^]^

It is worth noting that introducing magnetic nanoparticles into the hydrogel DDS can offer magnetic guidance for delivering drugs at targeted areas, thereby achieved non‐contact drug targeted delivery.^[^ ^279^ ^]^ Thus, an SA hydrogel‐based magnetic spring comprising magnetically aligned Fe_2_O_3_ NPs, was fabricated by Zheng et al. The hydrogel displayed magnetic locomotion and active heating capacities, making them suitable for targeted magnetic hyperthermia. In the high‐frequency alternating magnetic field condition, a temperature of 43 °C was achieved and resulted in shrinkage, thus leading to 35% higher drug release than at 37 °C.^[^ ^272^ ^]^ In the work of Liao and Huang, a dual pH/magnetic‐sensitive hydrogel was fabricated via in situ formation of Fe_3_O_4_ inside carboxymethyl chitin network. The fabricated hydrogels could be attracted by the magnet, and sustainably shrink at low pH to promote 5‐fluorouracil release in SGF.^[^ ^273^ ^]^

The efficacy of shrinkable DDS in sustainably releasing biomacromolecules, has been determined by Song et al. and Ding et al. The former team synthesized self‐healing hydrogels with thermo‐ and reduction‐responsiveness through photo‐crosslinking of PF127 end functionalized with α‐lipoic acid. After loading BSA into the hydrogels, applying a reducing agent could accelerate the BSA release rate, while the thermo‐responsive volume shrinkage could inhibit the BSA burst release and improved sustained release behavior. Importantly, the hydrogels demonstrated no negative impact on metabolic process of L929 mouse fibroblast.^[^ ^267^ ^]^ Interestingly, the latter group preserved the pH‐responsiveness of CS by virtue of a protection/deprotection strategy to ‐NH_2_ during the process of introducing allyl groups into CS. Thiol modified 4‐arm PEG was exploited as crosslinker to realize fast gelation via thiol‐ene click reaction. The resulted hydrogel displayed good swelling behavior in neutral and acidic liquids, but shrinkage behavior in basic liquids. This feature made the hydrogel considerably promote the release of small molecule drug DOX in basic liquids, while delay the release of macromolecule drug BSA. And these results strongly verified the opposite release behavior of small molecule drug and macromolecule drug in the volume shrinkage process of SHs DDS.^[^ ^280^ ^]^

Collectively, SHs have offered efficient strategies for controlled drug delivery owing to its stimuli‐responsive volume shrinkage behavior. However, recent studies have scarcely determined the in vivo on‐demand drug release of SHs, which impeded the practical application of SHs DDS. In addition, comprehensive biological studies should be performed to verify the in vivo safety of SHs and applied extrinsic stimulus. We firmly believe that SHs DDS will hold an important position in biomedical fields of controlled and localized delivery of drugs in the foreseeable future.

## 5 Conclusions and Perspectives

Over the past five years, hydrogels are experiencing vigorous development and have been broadly utilized in biomedical areas. For the first time, the recent advances of hydrogels with different swelling features were reviewed comprehensively. And in this review, high‐swelling hydrogels, non‐swelling hydrogels, and shrinkable hydrogels are successively generalized and analyzed from two aspects: constructions and biomedical applications.

HSHs refer to a type of hydrogels which have excellent swelling capacity in liquid media. They can be constructed based on natural polymers, synthetic polymers, or mixtures of both. NSHs are hydrogels whose swelling behavior is very nearly an equilibrium state. Thus, they can retain their initial shapes and intrinsic properties in physiological environment even for a prolonged time. Common strategies on developing NSHs focus on increasing the cross‐linking density, regulating the polymer/water interaction, or introducing thermosensitive polymers. SHs are a library of stimuli‐responsive hydrogels that can reversibly deswell (i.e., shrink) with changes in external conditions, like temperature, or pH. Notably, most SHs are achieved by incorporating thermo‐responsive polymers. All the aforementioned hydrogels are highly appreciated in a myriad of biomedical fields. Concretely, in tissue engineering and regenerative medicine, HSHs show great prospects for full‐thickness skin wound healing. But in fields of tissue adhesives and internal wound healing, the application of NSHs can perfectly avoid the possible adverse effects brought from swelling. In addition, SHs have recently emerged for skin wound healing as they can concurrently drive wound closure and enhance wound healing. And the shrinkage behavior has also been used to achieve resolution enhancement of hydrogel scaffolds. Furthermore, both HSHs and SHs played very significant functions in drug delivery with different drug release mechanisms. Specially, the non‐swelling ability of conductive NSHs make them excellent bioelectronic devices for meeting the real‐time monitoring of human motions and bioelectric.

However, there are still several difficulties that remain to be addressed for broadening their applications. For instance, the suitability of hydrogel wound dressings (especially SHs) in burn injuries or diabetic chronic wounds have rarely been confirmed, which set higher demands in the biofunctions of the dressings. The wound treatment to delicate skin faces of babies, children, and elderly people have been ignored as well. Furthermore, it is advantageous to render the degradation rate of hydrogel scaffolds match the rate of tissue regeneration. Despite of the proved high drug loading efficiency and sustained in vitro release profile of HSHs and SHs DDS, there is an urgent need to strongly verify their in vivo safety and therapeutic effects for common clinical practice. In addition, detailed comparisons should be drawn between the two drug release mechanisms in order to achieve better localized drug delivery. Overall, we steadily believe hydrogels with different swelling behaviors will have better performance and wider biomedical applications in the foreseeable future.

## Conflict of Interest

The authors declare no conflict of interest.

## Author Contributions

Z.W. proposed the topic of the review. W.F. investigated the literature and prepared the manuscript. Z.W. revised the manuscript.
